# Supplementary material for: Cellomics approach for high-throughput functional annotation of Caenorhabditis elegans neural network
Source: Sci Rep. 2018 Jul 10;8:10380. doi: 10.1038/s41598-018-28653-x (PMC6039433; doi:10.1038/s41598-018-28653-x)
Supplement: Supplementary file 3 — Supplementary file 2 [file 41598_2018_28653_MOESM3_ESM.pdf]

Cellomics approach for high-throughput functional annotation of *Caenorhabditis elegans* neural network

Wataru Aoki[1,2,3,*], Hidenori Matsukura[1], Yuji Yamauchi[1], Haruki Yokoyama[1], Koichi Hasegawa[4], Ryoji Shinya[5], Mitsuyoshi Ueda[1,3]

[1]Division of Applied Life Sciences, Graduate School of Agriculture, Kyoto University, Sakyo-ku, Kyoto 606-8502, Japan

[2]JST, PRESTO, 4-1-8 Honcho, Kawaguchi, Saitama 332-0012, Japan

[3]Kyoto Integrated Science & Technology Bio-Analysis Center, Shimogyo-ku, Kyoto 600-8813, Japan

[4]Department of Environmental Biology, College of Bioscience and Biotechnology, Chubu University, Kasugai 487-8501, Japan

[5]Department of Agriculture, School of Agriculture, Meiji University, Tama-ku, Kawasaki 214-8571, Japan

*Correspondence should be addressed to: Wataru Aoki

Tel.: +81-75-753-6495; Fax: +81-75-753-6112; E-mail: aoki.wataru.6a@kyoto-u.ac.jp

pCre (4838 bp)

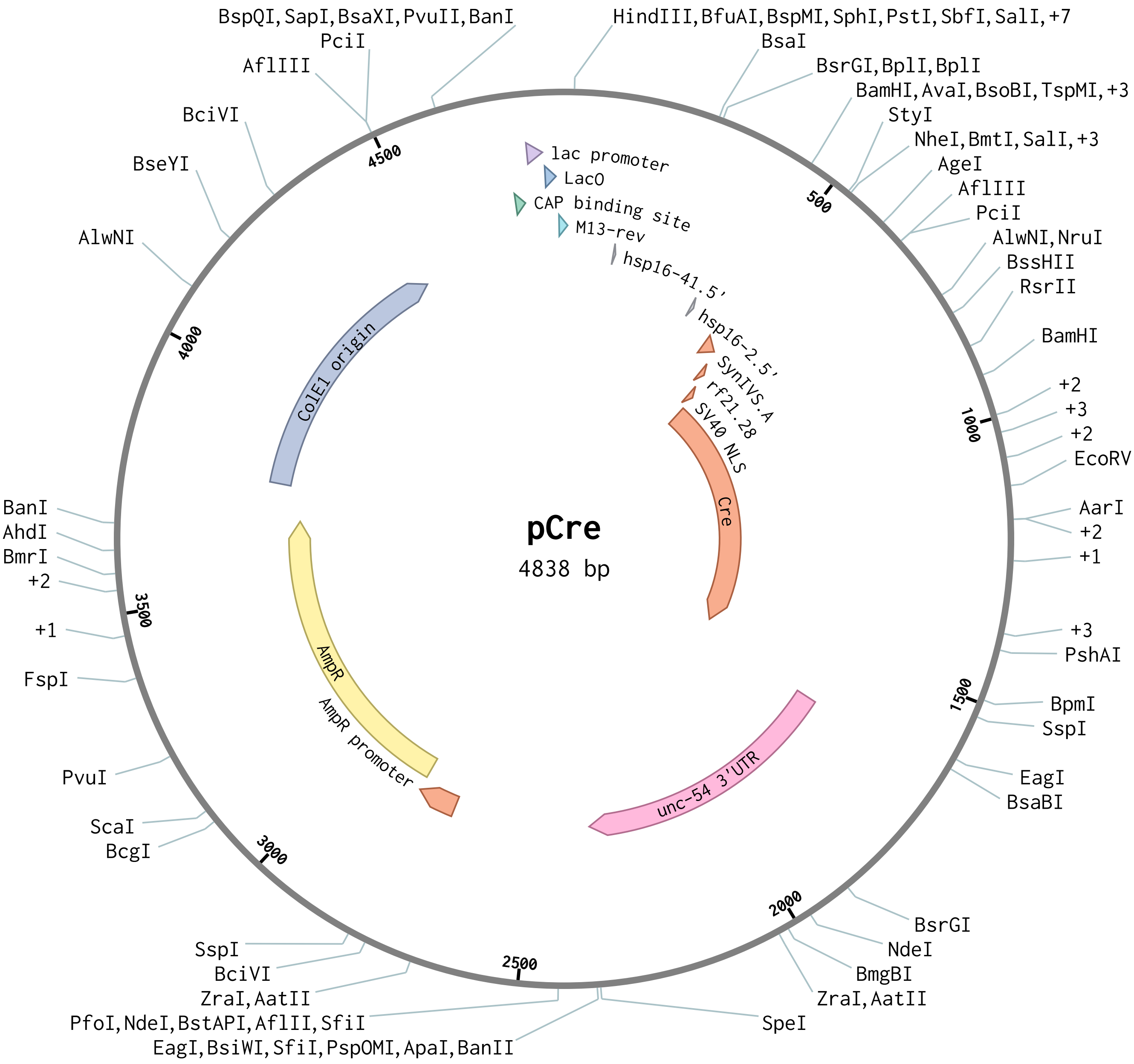
pCre
4838 bp
HindIII,BfuAI,BspMI,SphI,PstI,SbfI,SalI,+7
BsaI
BsrGI,BplI,BplI
BamHI,AvaI,BsoBI,TspMI,+3
StyI
NheI,BmtI,SalI,+3
AgeI
AflIII
PciI
AlwNI,NruI
BssHII
RsrII
BamHI
+2
+3
+2
EcoRV
AarI
+2
+1
+3
PshAI
BpmI
SspI
EagI
BsaBI
BsrGI
NdeI
BmgBI
ZraI,AatII
SpeI
EagI,BsiWI,SfiI,PspOMI,ApaI,BanII
PfoI,NdeI,BstAPI,AflII,SfiI
ZraI,AatII
BciVI
SspI
BcgI
ScaI
PvuI
FspI
+1
+2
BmrI
AhdI
BanI
AlwNI
BseYI
BciVI
AflIII
PciI
BspQI,SapI,BsaXI,PvuII,BanI
lac promoter
LacO
CAP binding site
M13-rev
hsp16-41.5'
hsp16-2.5'
SynIVS.A
rf21.28
SV40 NLS
Cre
unc-54 3'UTR
AmpR
AmpR promoter
ColE1 origin
500
1000
1500
2000
2500
3000
3500
4000
4500

pCre (4838 bp)

HincII
AccI
SalI
BspMI
BfuAI
SbfI
HindIII SphI PstI

ATGACCATGATTACGCCAAGCTTGCATGCCTGCAGGTCGACTCTAGAGGATCAAGAGCATTTGAATCAGAATATGGAGAACGGAGCATGAGCATTTTCGAAGTTTTT
TACTGGTACTAATGCGGTTCGAACGTACGGACGTCCAGCTGAGATCTCCTAGTTCTCGTAAACTTAGTCTTATACCTCTTGCCTCGTACTCGTAAAAGCTTCAAAAA

20 40 60 80 100

NsiI
BanII BsmI PstI BsmI

TAGATGCACTAGAACAAAGCGTGTTGGCTTCCTCTGAGCCCGCTTTCCTTATATACCCGCATTCTGCAGCCTTACAGAATGTTCTAGAAGGTCCTAGATGCATTCGT
ATCTACGTGATCTTGTTTCGCACAACCGAAGGAGACTCGGGCGAAAGGAATATATGGGCGTAAGACGTCGGAATGTCTTACAAGATCTTCCAGGATCTACGTAAGCA

120 140 160 180 200

BsaI BsrGI BplI

TTGAAAATACTCCCGGTGGGTGCAAAGAGACGCAGACGGAAAATGTATCTGGGTCTCTTTATTGTGTACACTACTTTTCCATGTACCGAATGTGAGTCGCCCTCCTT
AACTTTTATGAGGGCCACCCACGTTTCTCTGCGTCTGCCTTTTACATAGACCCAGAGAAATAACACATGTGATGAAAAGGTACATGGCTTACACTCAGCGGGAGGAA

220 240 260 280 300 320

BplI

TTGCAACAAGCAGCTCGAATGTTCTAGAAAAAGGTGGAAAATAGTATAAATACCGTTGAAAATAAATACCGAACAACATTTGCTCTAATTGTGAAATTAGAAATCTT
AACGTTGTTCGTCGAGCTTACAAGATCTTTTTCCACCTTTTATCATATTTATGGCAACTTTTATTTATGGCTTGTTGTAAACGAGATTAACACTTTAATCTTTAGAA

340 360 380 400 420

TspMI
BsoBI
XmaI
AvaI NheI
BamHI SmaI MscI StyI

CAAACTATAATCATctcactgGATCCCCGGGATTGGCCAAAGGACCCAAAGGTATGTTTCGAATGATACTAACATAACATAGAACATTTTCAGGAGGACCCTTGGCT
GTTTGATATTAGTAgagtgacCTAGGGGCCCTAACCGGTTTCCTGGGTTTCCATACAAAGCTTACTATGATTGTATTGTATCTTGTAAAAGTCCTCCTGGGAACCGA

SynIVS.A

440 460 480 500 520

HincII
AccI
SgrDI
SalI
BmtI
AgeI

AGCGTCGACGATGCCCAAGAAGAAGAGGAAGGTGTCCAATTTACTGACCGTACACCAAAATTTGCCTGCATTACCGGTCGATGCAACGAGTGATGAGGTTCGCAAGA
TCGCAGCTGCTACGGGTTCTTCTTCTCCTTCCACAGGTTAAATGACTGGCATGTGGTTTTAAACGGACGTAATGGCCAGCTACGTTGCTCACTACTCCAAGCGTTCT

SV40 NLS Cre

540 560 580 600 620 640

AflIII
PciI

ACCTGATGGACATGTTCAGGGATCGCCAGGCGTTTTCTGAGCATACCTGGAAAATGCTTCTGTCCGTTTGCCGGTCGTGGGCGGCATGGTGCAAGTTGAATAACCGG
TGGACTACCTGTACAAGTCCCTAGCGGTCCGCAAAAGACTCGTATGGACCTTTTACGAAGACAGGCAAACGGCCAGCACCCGCCGTACCACGTTCAACTTATTGGCC

Cre

660 680 700 720 740

AlwNI NruI BssHII

AAATGGTTTCCCGCAGAACCTGAAGATGTTCGCGATTATCTTCTATATCTTCAGGCGCGCGGTCTGGCAGTAAAAACTATCCAGCAACATTTGGGCCAGCTAAACAT
TTTACCAAAGGGCGTCTTGGACTTCTACAAGCGCTAATAGAAGATATAGAAGTCCGCGCGCCAGACCGTCATTTTTGATAGGTCGTTGTAAACCCGGTCGATTTGTA

Cre

760 780 800 820 840

RsrII BamHI

GCTTCATCGTCGGTCCGGGCTGCCACGACCAAGTGACAGCAATGCTGTTTCACTGGTTATGCGGCGGATCCGAAAAGAAAACGTTGATGCCGGTGAACGTGCAAAAC
CGAAGTAGCAGCCAGGCCCGACGGTGCTGGTTCACTGTCGTTACGACAAAGTGACCAATACGCCGCCTAGGCTTTTCTTTTGCAACTACGGCCACTTGCACGTTTTG

Cre

860 880 900 920 940 960

AsiSI
SexAI BcgI PvuI SnaBI PsiI

AGGCTCTAGCGTTCGAACGCACTGATTTCGACCAGGTTCGTTCACTCATGGAAAATAGCGATCGCTGCCAGGATATACGTAATCTGGCATTTCTGGGGATTGCTTAT
TCCGAGATCGCAAGCTTGCGTGACTAAAGCTGGTCCAAGCAAGTGAGTACCTTTTATCGCTAGCGACGGTCCTATATGCATTAGACCGTAAAGACCCCTAACGAATA

Cre

980 1,000 1,020 1,040 1,060

AarI
BspMI
SnaBI EcoRV BfuAI

AACACCCTGTTACGTATAGCCGAAATTGCCAGGATCAGGGTTAAAGATATCTCACGTACTGACGGTGGGAGAATGTTAATCCATATTGGCAGAACGAAAACGCTGGT
TTGTGGGACAATGCATATCGGCTTTAACGGTCCTAGTCCCAATTTCTATAGAGTGCATGACTGCCACCCTCTTACAATTAGGTATAACCGTCTTGCTTTTGCGACCA

Cre

1,080 1,100 1,120 1,140 1,160

BtgZI

TAGCACCGCAGGTGTAGAGAAGGCACTTAGCCTGGGGGTAACTAAACTGGTCGAGCGATGGATTTCCGTCTCTGGTGTAGCTGATGATCCGAATAACTACCTGTTTT
ATCGTGGCGTCCACATCTCTTCCGTGAATCGGACCCCCATTGATTTGACCAGCTCGCTACCTAAAGGCAGAGACCACATCGACTACTAGGCTTATTGATGGACAAAA

Cre

1,180 1,200 1,220 1,240 1,260 1,280

BspDI ClaI Bpu10I

GCCGGGTCAGAAAAAATGGTGTTGCCGCGCCATCTGCCACCAGCCAGCTATCAACTCGCGCCCTGGAAGGGATTTTTGAAGCAACTCATCGATTGATTTACGGCGCT
CGGCCCAGTCTTTTTTACCACAACGGCGCGGTAGACGGTGGTCGGTCGATAGTTGAGCGCGGGACCTTCCCTAAAAACTTCGTTGAGTAGCTAACTAAATGCCGCGA

Cre

1,300 1,320 1,340 1,360 1,380

PshAI BpmI

AAGGATGACTCTGGTCAGAGATACCTGGCCTGGTCTGGACACAGTGCCCGTGTCGGAGCCGCGCGAGATATGGCCCGCGCTGGAGTTTCAATACCGGAGATCATGCA
TTCCTACTGAGACCAGTCTCTATGGACCGGACCAGACCTGTGTCACGGGCACAGCCTCGGCGCGCTCTATACCGGGCGCGACCTCAAAGTTATGGCCTCTAGTACGT

Cre

1,400 1,420 1,440 1,460 1,480

SspI EagI

AGCTGGTGGCTGGACCAATGTAAATATTGTCATGAACTATATCCGTAACCTGGATAGTGAAACAGGGGCAATGGTGCGCCTGCTGGAAGATGGCGATTAGCGCATCG
TCGACCACCGACCTGGTTACATTTATAACAGTACTTGATATAGGCATTGGACCTATCACTTTGTCCCCGTTACCACGCGGACGACCTTCTACCGCTAATCGCGTAGC

Cre

1,500 1,520 1,540 1,560 1,580 1,600

BsaBI

GCCGCTGTCATCAGATCGCCATCTCGCGCCCGTGCCTCTGACTTCTAAGTCCAATTACTCTTCAACATCCCTACATGCTCTTTCTCCCTGTGCTCCCACCCCCTATT
CGGCGACAGTAGTCTAGCGGTAGAGCGCGGGCACGGAGACTGAAGATTCAGGTTAATGAGAAGTTGTAGGGATGTACGAGAAAGAGGGACACGAGGGTGGGGGATAA

unc-54 3'UTR

1,620 1,640 1,660 1,680 1,700

TTTGTTATTATCAAAAAACTTCTCTTAATTTCTTTGTTTTTTAGCTTCTTTTAAGTCACCTCTAACAATGAAATTGTGTAGATTCAAAAATAGAATTAATTCGTAAT
AAACAATAATAGTTTTTTGAAGAGAATTAAAGAAACAAAAAATCGAAGAAAATTCAGTGGAGATTGTTACTTTAACACATCTAAGTTTTTATCTTAATTAAGCATTA

unc-54 3'UTR

1,720 1,740 1,760 1,780 1,800

BsrGI

AAAAAGTCGAAAAAAATTGTGCTCCCTCCCCCCATTAATAATAATTCTATCCCAAAATCTACACAATGTTCTGTGTACACTTCTTATGTTTTTTACTTCTGATAAAT
TTTTTCAGCTTTTTTTAACACGAGGGAGGGGGGTAATTATTATTAAGATAGGGTTTTAGATGTGTTACAAGACACATGTGAAGAATACAAAAAATGAAGACTATTTA

unc-54 3'UTR

1,820 1,840 1,860 1,880 1,900 1,920

NdeI BmgBI

TTTTTTGAAACATCATAGAAAAAACCGCACACAAAATACCTTATCATATGTTACGTTTCAGTTTATGACCGCAATTTTTATTTCTTCGCACGTCTGGGCCTCTCATG
AAAAAACTTTGTAGTATCTTTTTTGGCGTGTGTTTTATGGAATAGTATACAATGCAAAGTCAAATACTGGCGTTAAAAATAAAGAAGCGTGCAGACCCGGAGAGTAC

unc-54 3'UTR

1,940 1,960 1,980 2,000 2,020

AatII
ZraI

ACGTCAAATCATGCTCATCGTGAAAAAGTTTTGGAGTATTTTTGGAATTTTTCAATCAAGTGAAAGTTTATGAAATTAATTTTCCTGCTTTTGCTTTTTGGGGTTTC
TGCAGTTTAGTACGAGTAGCACTTTTTCAAAACCTCATAAAAACCTTAAAAAGTTAGTTCACTTTCAAATACTTTAATTAAAAGGACGAAAACGAAAAACCCCAAAG

unc-54 3'UTR

2,040 2,060 2,080 2,100 2,120 2,140

CCCTATTGTTTGTCAAGATTTCGAGGACGGCGTTTTTCTTGCTAAAATCACAAGTATTGATGAGCACGATGCAAGAAAGATCGGAAGAAGGTTTGGGTTTGAGGCTC
GGGATAACAAACAGTTCTAAAGCTCCTGCCGCAAAAAGAACGATTTTAGTGTTCATAACTACTCGTGCTACGTTCTTTCTAGCCTTCTTCCAAACCCAAACTCCGAG

unc-54 3'UTR

2,160 2,180 2,200 2,220 2,240

AGTGGAAGGTGAGTAGAAGTTGATAATTTGAAAGTGGAGTAGTGTCTATGGGGTTTTTGCCTTAAATGACAGAATACATTCCCAATATACCAAACATAACTGTTTCC
TCACCTTCCACTCATCTTCAACTATTAAACTTTCACCTCATCACAGATACCCCAAAAACGGAATTTACTGTCTTATGTAAGGGTTATATGGTTTGTATTGACAAAGG

unc-54 3'UTR

2,260 2,280 2,300 2,320 2,340

BanII
PspOMI
BsiWI ApaI
SpeI EagI SfiI PfoI

TACTAGTCGGCCGTACGGGCCCTTTCGTCTCGCGCGTTTCGGTGATGACGGTGAAAACCTCTGACACATGCAGCTCCCGGAGACGGTCACAGCTTGTCTGTAAGCGG
ATGATCAGCCGGCATGCCCGGGAAAGCAGAGCGCGCAAAGCCACTACTGCCACTTTTGGAGACTGTGTACGTCGAGGGCCTCTGCCAGTGTCGAACAGACATTCGCC

2,360 2,380 2,400 2,420 2,440 2,460

NdeI

ATGCCGGGAGCAGACAAGCCCGTCAGGGCGCGTCAGCGGGTGTTGGCGGGTGTCGGGGCTGGCTTAACTATGCGGCATCAGAGCAGATTGTACTGAGAGTGCACCAT
TACGGCCCTCGTCTGTTCGGGCAGTCCCGCGCAGTCGCCCACAACCGCCCACAGCCCCGACCGAATTGATACGCCGTAGTCTCGTCTAACATGACTCTCACGTGGTA

2,480 2,500 2,520 2,540 2,560

SfiI
BstAPI AflII

ATGCGGTGTGAAATACCGCACAGATGCGTAAGGAGAAAATACCGCATCAGGCGGCCTTAAGGGCCTCGTGATACGCCTATTTTTATAGGTTAATGTCATGATAATAA
TACGCCACACTTTATGGCGTGTCTACGCATTCCTCTTTTATGGCGTAGTCCGCCGGAATTCCCGGAGCACTATGCGGATAAAAATATCCAATTACAGTACTATTATT

2,580 2,600 2,620 2,640 2,660

AatII
ZraI
BciVI

TGGTTTCTTAGACGTCAGGTGGCACTTTTCGGGGAAATGTGCGCGGAACCCCTATTTGTTTATTTTTCTAAATACATTCAAATATGTATCCGCTCATGAGACAATAA
ACCAAAGAATCTGCAGTCCACCGTGAAAAGCCCCTTTACACGCGCCTTGGGGATAAACAAATAAAAAGATTTATGTAAGTTTATACATAGGCGAGTACTCTGTTATT

AmpR promoter

2,680 2,700 2,720 2,740 2,760 2,780

SspI

CCCTGATAAATGCTTCAATAATATTGAAAAAGGAAGAGTATGAGTATTCAACATTTCCGTGTCGCCCTTATTCCCTTTTTTGCGGCATTTTGCCTTCCTGTTTTTGC
GGGACTATTTACGAAGTTATTATAACTTTTTCCTTCTCATACTCATAAGTTGTAAAGGCACAGCGGGAATAAGGGAAAAAACGCCGTAAAACGGAAGGACAAAAACG

AmpR promoter AmpR

2,800 2,820 2,840 2,860 2,880

TCACCCAGAAACGCTGGTGAAAGTAAAAGATGCTGAAGATCAGTTGGGTGCACGAGTGGGTTACATCGAACTGGATCTCAACAGCGGTAAGATCCTTGAGAGTTTTC
AGTGGGTCTTTGCGACCACTTTCATTTTCTACGACTTCTAGTCAACCCACGTGCTCACCCAATGTAGCTTGACCTAGAGTTGTCGCCATTCTAGGAACTCTCAAAAG

AmpR

2,900 2,920 2,940 2,960 2,980

GCCCCGAAGAACGTTTTCCAATGATGAGCACTTTTAAAGTTCTGCTATGTGGCGCGGTATTATCCCGTATTGACGCCGGGCAAGAGCAACTCGGTCGCCGCATACAC
CGGGGCTTCTTGCAAAAGGTTACTACTCGTGAAAATTTCAAGACGATACACCGCGCCATAATAGGGCATAACTGCGGCCCGTTCTCGTTGAGCCAGCGGCGTATGTG

AmpR

3,000 3,020 3,040 3,060 3,080 3,100

BcgI ScaI

TATTCTCAGAATGACTTGGTTGAGTACTCACCAGTCACAGAAAAGCATCTTACGGATGGCATGACAGTAAGAGAATTATGCAGTGCTGCCATAACCATGAGTGATAA
ATAAGAGTCTTACTGAACCAACTCATGAGTGGTCAGTGTCTTTTCGTAGAATGCCTACCGTACTGTCATTCTCTTAATACGTCACGACGGTATTGGTACTCACTATT

AmpR

3,120 3,140 3,160 3,180 3,200

PvuI

CACTGCGGCCAACTTACTTCTGACAACGATCGGAGGACCGAAGGAGCTAACCGCTTTTTTGCACAACATGGGGGATCATGTAACTCGCCTTGATCGTTGGGAACCGG
GTGACGCCGGTTGAATGAAGACTGTTGCTAGCCTCCTGGCTTCCTCGATTGGCGAAAAAACGTGTTGTACCCCCTAGTACATTGAGCGGAACTAGCAACCCTTGGCC

AmpR

3,220 3,240 3,260 3,280 3,300

FspI

AGCTGAATGAAGCCATACCAAACGACGAGCGTGACACCACGATGCCTGTAGCAATGGCAACAACGTTGCGCAAACTATTAACTGGCGAACTACTTACTCTAGCTTCC
TCGACTTACTTCGGTATGGTTTGCTGCTCGCACTGTGGTGCTACGGACATCGTTACCGTTGTTGCAACGCGTTTGATAATTGACCGCTTGATGAATGAGATCGAAGG

AmpR

3,320 3,340 3,360 3,380 3,400 3,420

NmeAIII

CGGCAACAATTAATAGACTGGATGGAGGCGGATAAAGTTGCAGGACCACTTCTGCGCTCGGCCCTTCCGGCTGGCTGGTTTATTGCTGATAAATCTGGAGCCGGTGA
GCCGTTGTTAATTATCTGACCTACCTCCGCCTATTTCAACGTCCTGGTGAAGACGCGAGCCGGGAAGGCCGACCGACCAAATAACGACTATTTAGACCTCGGCCACT

AmpR

3,440 3,460 3,480 3,500 3,520

BsaI
BpmI BmrI AhdI

GCGTGGGTCTCGCGGTATCATTGCAGCACTGGGGCCAGATGGTAAGCCCTCCCGTATCGTAGTTATCTACACGACGGGGAGTCAGGCAACTATGGATGAACGAAATA
CGCACCCAGAGCGCCATAGTAACGTCGTGACCCCGGTCTACCATTCGGGAGGGCATAGCATCAATAGATGTGCTGCCCCTCAGTCCGTTGATACCTACTTGCTTTAT

AmpR

3,540 3,560 3,580 3,600 3,620

BanI

GACAGATCGCTGAGATAGGTGCCTCACTGATTAAGCATTGGTAACTGTCAGACCAAGTTTACTCATATATACTTTAGATTGATTTAAAACTTCATTTTTAATTTAAA
CTGTCTAGCGACTCTATCCACGGAGTGACTAATTCGTAACCATTGACAGTCTGGTTCAAATGAGTATATATGAAATCTAACTAAATTTTGAAGTAAAAATTAAATTT

AmpR

3,640 3,660 3,680 3,700 3,720 3,740

AGGATCTAGGTGAAGATCCTTTTTGATAATCTCATGACCAAAATCCCTTAACGTGAGTTTTCGTTCCACTGAGCGTCAGACCCCGTAGAAAAGATCAAAGGATCTTC
TCCTAGATCCACTTCTAGGAAAAACTATTAGAGTACTGGTTTTAGGGAATTGCACTCAAAAGCAAGGTGACTCGCAGTCTGGGGCATCTTTTCTAGTTTCCTAGAAG

ColE1 origin

3,760 3,780 3,800 3,820 3,840

TTGAGATCCTTTTTTTCTGCGCGTAATCTGCTGCTTGCAAACAAAAAAACCACCGCTACCAGCGGTGGTTTGTTTGCCGGATCAAGAGCTACCAACTCTTTTTCCGA
AACTCTAGGAAAAAAAGACGCGCATTAGACGACGAACGTTTGTTTTTTTGGTGGCGATGGTCGCCACCAAACAAACGGCCTAGTTCTCGATGGTTGAGAAAAAGGCT

ColE1 origin

3,860 3,880 3,900 3,920 3,940

AGGTAACTGGCTTCAGCAGAGCGCAGATACCAAATACTGTCCTTCTAGTGTAGCCGTAGTTAGGCCACCACTTCAAGAACTCTGTAGCACCGCCTACATACCTCGCT
TCCATTGACCGAAGTCGTCTCGCGTCTATGGTTTATGACAGGAAGATCACATCGGCATCAATCCGGTGGTGAAGTTCTTGAGACATCGTGGCGGATGTATGGAGCGA

ColE1 origin

3,960 3,980 4,000 4,020 4,040 4,060

AlwNI

CTGCTAATCCTGTTACCAGTGGCTGCTGCCAGTGGCGATAAGTCGTGTCTTACCGGGTTGGACTCAAGACGATAGTTACCGGATAAGGCGCAGCGGTCGGGCTGAAC
GACGATTAGGACAATGGTCACCGACGACGGTCACCGCTATTCAGCACAGAATGGCCCAACCTGAGTTCTGCTATCAATGGCCTATTCCGCGTCGCCAGCCCGACTTG

ColE1 origin

4,080 4,100 4,120 4,140 4,160

BseYI

GGGGGGTTCGTGCACACAGCCCAGCTTGGAGCGAACGACCTACACCGAACTGAGATACCTACAGCGTGAGCATTGAGAAAGCGCCACGCTTCCCGAAGGGAGAAAGG
CCCCCCAAGCACGTGTGTCGGGTCGAACCTCGCTTGCTGGATGTGGCTTGACTCTATGGATGTCGCACTCGTAACTCTTTCGCGGTGCGAAGGGCTTCCCTCTTTCC

ColE1 origin

4,180 4,200 4,220 4,240 4,260 4,280

BciVI

CGGACAGGTATCCGGTAAGCGGCAGGGTCGGAACAGGAGAGCGCACGAGGGAGCTTCCAGGGGGAAACGCCTGGTATCTTTATAGTCCTGTCGGGTTTCGCCACCTC
GCCTGTCCATAGGCCATTCGCCGTCCCAGCCTTGTCCTCTCGCGTGCTCCCTCGAAGGTCCCCCTTTGCGGACCATAGAAATATCAGGACAGCCCAAAGCGGTGGAG

ColE1 origin

4,300 4,320 4,340 4,360 4,380

TGACTTGAGCGTCGATTTTTGTGATGCTCGTCAGGGGGGCGGAGCCTATGGAAAAACGCCAGCAACGCGGCCTTTTTACGGTTCCTGGCCTTTTGCTGGCCTTTTGC
ACTGAACTCGCAGCTAAAAACACTACGAGCAGTCCCCCCGCCTCGGATACCTTTTTGCGGTCGTTGCGCCGGAAAAATGCCAAGGACCGGAAAACGACCGGAAAACG

ColE1 origin

4,400 4,420 4,440 4,460 4,480

AflIII
PciI

TCACATGTTCTTTCCTGCGTTATCCCCTGATTCTGTGGATAACCGTATTACCGCCTTTGAGTGAGCTGATACCGCTCGCCGCAGCCGAACGACCGAGCGCAGCGAGT
AGTGTACAAGAAAGGACGCAATAGGGGACTAAGACACCTATTGGCATAATGGCGGAAACTCACTCGACTATGGCGAGCGGCGTCGGCTTGCTGGCTCGCGTCGCTCA

4,500 4,520 4,540 4,560 4,580 4,600

BspQI
SapI BsaXI PvuII

CAGTGAGCGAGGAAGCGGAAGAGCGCCCAATACGCAAACCGCCTCTCCCCGCGCGTTGGCCGATTCATTAATGCAGCTGGCACGACAGGTTTCCCGACTGGAAAGCG
GTCACTCGCTCCTTCGCCTTCTCGCGGGTTATGCGTTTGGCGGAGAGGGGCGCGCAACCGGCTAAGTAATTACGTCGACCGTGCTGTCCAAAGGGCTGACCTTTCGC

4,620 4,640 4,660 4,680 4,700

BanI

GGCAGTGAGCGCAACGCAATTAATGTGAGTTAGCTCACTCATTAGGCACCCCAGGCTTTACACTTTATGCTTCCGGCTCGTATGTTGTGTGGAATTGTGAGCGGATA
CCGTCACTCGCGTTGCGTTAATTACACTCAATCGAGTGAGTAATCCGTGGGGTCCGAAATGTGAAATACGAAGGCCGAGCATACAACACACCTTAACACTCGCCTAT

CAP binding site lac promoter LacO

4,720 4,740 4,760 4,780 4,800

ACAATTTCACACAGGAAACAGCT
TGTTAAAGTGTGTCCTTTGTCGA

M13-rev

4,820 4,830

No_007_pPD49_78_F25B3.3p_mCherry (7584 bp)

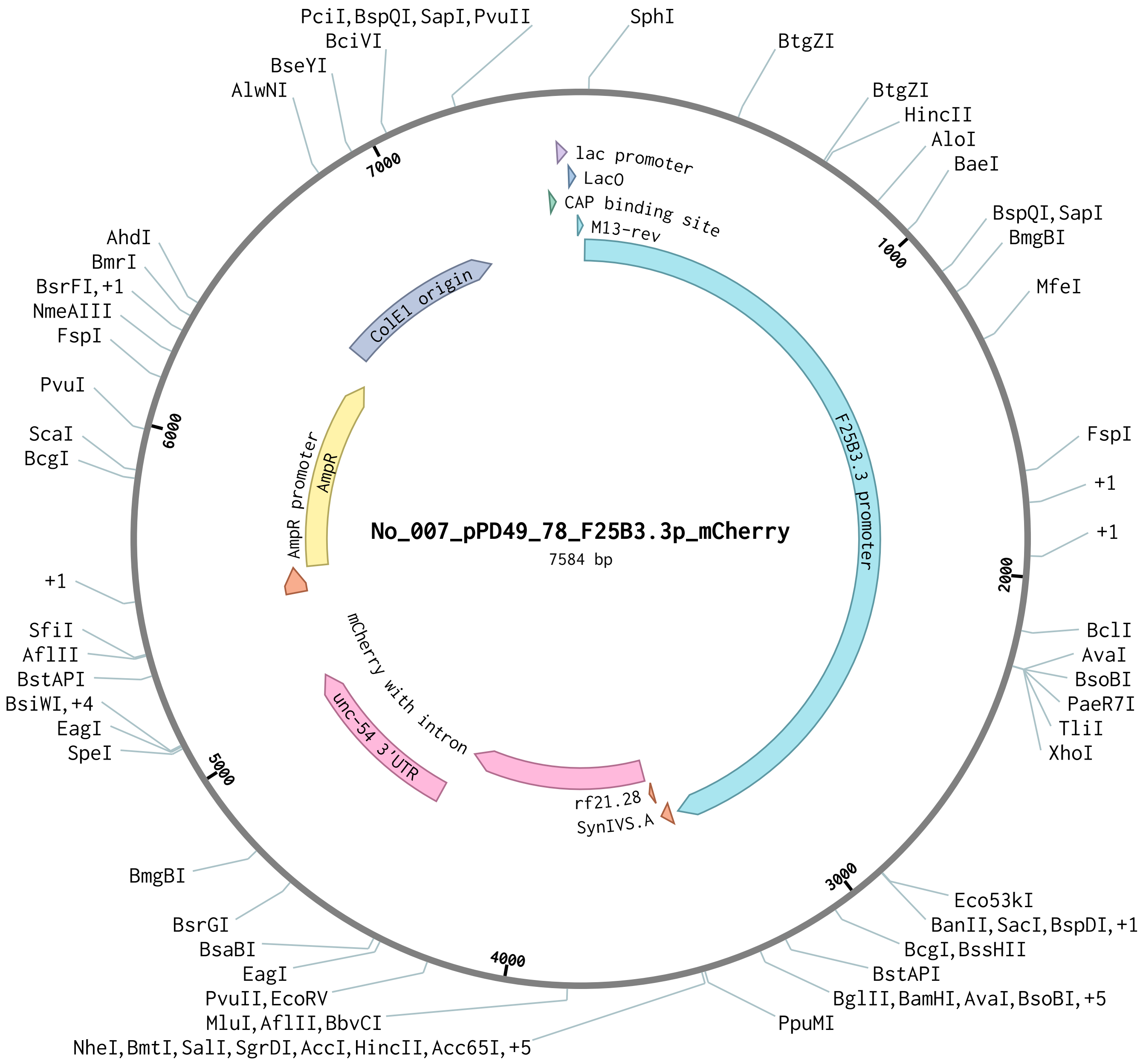

No_007_pPD49_78_F25B3.3p_mCherry (7584 bp)

SphI
ATGACCATGATTACGCCgcatgcaagactaattttcgattaacccgtaggggtgcaagactaatagagactgcaagactattagaggctgaaatactaattttcgta
TACTGGTACTAATGCGGcgtacgttctgattaaaagctaattgggcatccccacgttctgattatctctgacgttctgataatctccgactttatgattaaaagcat
F25B3.3 promoter
20 40 60 80 100

tgctcaataattttggaaattggcctatttttttgtagaaacttgataccgtttaaacaaggaaaaatacacacttttttaatatttcatcaataatttgaacgatttt
acgagttattaaaacctttaaccggataaaaaacatctttgaactatggcaaatttgttcctttttatgtgtgaaaaattataaagtagttattaaacttgctaaaa
F25B3.3 promoter
120 140 160 180 200

gtgattttaagttcaatttgcccaaaaaaagacaattttttctgacgttaccgcaaagtaatgctggtcaggcaaaaagagcggtgcaaaaatataagagactgcaat
cactaaaattcaagttaaacgggtttttttctgttaaaaagactgcaatggcgtttcattacgaccagtccgtttttctcgccacgtttttatattctctgacgtta
F25B3.3 promoter
220 240 260 280 300 320

actaatagaggaaatacggtaattgaattttagtgaaacttgcgacagttttcctcatttttttgttattccggcatacgagtgtggcatacgagtgaggtcatctt
tgattatctcctttatgccattaacttaaaatcactttgaacgctgtcaaaaggagtaaaaaaacaataaggccgtatgctcacaccgtatgctcactccagtagaa
F25B3.3 promoter
340 360 380 400 420

BtgZI
ttgtttcttccgtttcttcatcgcttttgaaaaaaaatgttgaagaaaacttcgtagccgatagccggataatgtttggcacggcgttccaacatatatcattggaa
aacaaagaaggcaaagaagtagcgaaaacttttttttacaacttcttttgaagcatcggctatcggcctattacaaaccgtgccgcaaggttgtatatagtaacctt
F25B3.3 promoter
440 460 480 500 520

atttttaagatttttgcgaaaaatcacattcttcacgatgagaacacgttattgaaggaatataaatcaagaataacatatagttatatttctctattactttaac
taaaaaattctaaaaacgctttttagtgtaagaagtgctactcttgtgcaataacttccttatatttagttcttattgtatatcaatataaagagataatgaaattg
F25B3.3 promoter
540 560 580 600 620 640

BtgZI HincII
gttaaatatgagcaaatttgagcattttgattgcgatgaaaagcagaatcgagtcaactgaaatccgttcaaaaacctaagctcggttcgcccgaaagtcgatttttt
caatttatactcgtttaaactcgtaaaactaacgctacttttcgtcttagctcagttgactttaggcaagtttttggattcgagccaagcgggctttcagctaaaaa
F25B3.3 promoter
660 680 700 720 740

```
ccaatagccgaacagcactgcttctttattttccaggtatttttggggtgtaagaattatatcttctttgtttgggtatttacgtttccgaattccctttcttgatc
ggttatcggcttgtcgtgacgaagaaataaaaggtccataaaaaccccacattcttaatatagaagaaacaaacccataaatgcaaaggcttaagggaaagaactag
F25B3.3 promoter
760 780 800 820 840

AloI
tgtcgctatttttcagaaaccagaacagtttatcctttttcatgagaacctgaatagctcaaaaacccgtttaatttcttttcatcatctcatttgaactttccta
acagcgataaaaagtctttggtcttgtcaaataggaaaaagtactcttggacttatcgagtttttgggcaaattaaagaaaagtagtagagtaaactttgaaaggat
F25B3.3 promoter
860 880 900 920 940 960

BaeI
gtcttcttaatgatttccaggctcctcctacatttgtatctcaagatgcatacactttatttccgctccaattcgaatcatctacttctctttttttcttcaacttc
cagaagaattactaaaggtccgaggaggatgtaaacatagagttctacgtatgtgaaataaaggcgaggttaagcttagtagatgaagagaaaaaagaagttgaaag
F25B3.3 promoter
980 1,000 1,020 1,040 1,060

BspQI
SapI
tactttttttccattcttttctttcaaatgttcgacgtcttcaagtgactgctcttcattctcctcatttttctcgaatttcattcttgtctctttttttgctcaaaaa
atgaaaaaaggtaagaaaagaaagtttacaagctgcagaagttcactgacgagaagtaagaggagtaaaaagagcttaaagtaagaacagagaaaaaacgagttttt
F25B3.3 promoter
1,080 1,100 1,120 1,140 1,160

BmgBI
tggaaaatgaaagtgacgtgagatttggacggcgggacacgggggcagtagaagcagcaaaaaggagagaaagaggacacaaataagaagaacgaattcaaaaataa
accttttactttcactgcactctaaacctgccgccctgtgcccccgtcatcttcgtcgtttttcctctctttctcctgtgtttattcttcttgcttaagtttttatt
F25B3.3 promoter
1,180 1,200 1,220 1,240 1,260 1,280

MfeI
gcggagaggagctatttccgtcaattctacctccccaatcttcatcaattcggctcaattgaatgacgtcacagggagataaaccgtttggatgagcgccgtccatc
cgcctctcctcgataaaggcagttaagatggaggggttagaagtagttaagccgagttaacttactgcagtgtccctctatttggcaaacctactcgcggcaggtag
F25B3.3 promoter
1,300 1,320 1,340 1,360 1,380

actgacgccatcccgtttgggacaagaaaagagaaaaaagagcacaaagtttttggtgacggatcttgtcaatcatatgaaagttgttctgattgattgtcagtttt
tgactgcggtagggcaaaccctgttcttttctcttttttctcgtgtttcaaaaaccactgcctagaacagttagtatactttcaacaagactaactaacagtcaaaa
F25B3.3 promoter
1,400 1,420 1,440 1,460 1,480
```

tttcctactttttttggattctatccacttctgaacttttgacaagtttcaaactttctgaatcattttctatgcattttcctggaattctttttatgtaaaatatga
aaaggatgaaaaaacctaagataggtgaagacttgaaaactgttcaaagtttgaaagacttagtaaaagatacgtaaaaggaccttaagaaaaatacattttatact
F25B3.3 promoter
1,500 1,520 1,540 1,560 1,580 1,600

aatagaatgtttttgaattcaagttctgctttttttcttctttttttgttctgttcgggcttgggtatgcttttttttaaaaattattttgcacatcgaccaataagtgc
ttatcttacaaaaacttaagttcaagacgaaaaaagaagaaaaaacaagacaagcccgaacccatacgaaaaaaatttttaataaaacgtgtagctggttattcacg
F25B3.3 promoter
1,620 1,640 1,660 1,680 1,700

FspI BsaAI
gcaacttataaaattaatttatttttgttaattttttgaaatacttgtattgctttaagtgatctgacctcgccctgagctttccacgtagttatcaaatacaaatcc
cgttgaatattttaattaaataaaaacaattaaaaactttatgaacataacgaaattcactagactggagcgggactcgaaaggtgcatcaatagtttatgtttagg
F25B3.3 promoter
1,720 1,740 1,760 1,780 1,800

tccaagggtaacgtacctatattactgatctttataataactttatcacctgtccagttccagaggaattctgttaagcttataggcacagaaggagtcatttctgc
aggttcccattgcatggatataatgactagaaatattattgaaatagtggacaggtcaaggtctccttaagacaattcgaatatccgtgtcttcctcagtaaagacg
F25B3.3 promoter
1,820 1,840 1,860 1,880 1,900 1,920

BsaBI
tggtttttaatgatccaaatctttatttcaagtaaaaaactgaacacttgcgaataaaactatcagattaaccattcaccaaaaatgtgtttgaatctaaaactttc
accaaaaattactaggtttagaaataaagttcatttttttgacttgtgaacgcttattttgatagtctaattggtaagtggtttttacacaaacttagattttgaaag
F25B3.3 promoter
1,940 1,960 1,980 2,000 2,020

tcagtattccaaatatagaaataaataaccacgacattgctaaaatctgtctgaattgtgtactccttaccgtgaagtaataatggatataatgaatcgtttgaaat
agtcataaggtttatatctttatttattggtgctgtaacgattttagacagacttaacacatgaggaatggcacttcattattacctatattacttagcaaacttta
F25B3.3 promoter
2,040 2,060 2,080 2,100 2,120 2,140

XhoI
TliI
AvaI
BsoBI
BclI PaeR7I
gaatgatcagcacattttggtgaaagatcacaaataaggaataagcgacggaaaataaaacgattatttcggatcaaaaatttgttgaagatcatatacactcgag
cttactagtcgtgtaaaaccactttctagtgtttattccttattcgctgccttttattttgctaataaagcctagtttttaaacaacttctagtatatgtgagctc
F25B3.3 promoter
2,160 2,180 2,200 2,220 2,240

```
accaagattattctagacaattttcaaattggcttctttgtttgcaaatcttcataataatctgtgaagtttggaaatttgaatttttaatctttttttcatagatta
tggttctaataagatctgttaaaagtttaaccgaagaaacaaacgtttagaagtattattagacacttcaaacctttaaacttaaaaattagaaaaaagtatctaat
                                        F25B3.3 promoter
        2,260          2,280          2,300          2,320          2,340

tagtttttatttctttgcaaaactatattaaaaaccgatgcattgttttagggaaattaatgagccttttgttcaacactaaaaacaataaaattaaaattttggct
atcaaaaataaagaaacgttttgatataatttttggctacgtaacaaaatccctttaattactcggaaaacaagttgtgatttttgttattttaattttaaaaccga
                                        F25B3.3 promoter
   2,360          2,380          2,400          2,420          2,440          2,460

tcatcatttgaccttttttaagttcgaaaacttttctcgtatttctgaaccgccaatttttttcacacatctctagacttttggtgcccgttccagaaagttaagtaa
agtagtaaactggaaaaaattcaagcttttgaaaagagcataaagacttggcggttaaaaaaagtgtgtagagatctgaaaaccacgggcaaggtctttcaattcatt
                                        F25B3.3 promoter
            2,480          2,500          2,520          2,540          2,560

ttgctattctaagaaagttctcaacattgtttttttagttctgattgaattctgatgttccaggaatatattttaaatttaatattctttgcactatttctatactaa
aacgataagattctttcaagagttgtaacaaaaaaatcaagactaacttaagactacaaggtccttatataaaatttaaattataagaaacgtgataaagatatgatt
                                        F25B3.3 promoter
       2,580          2,600          2,620          2,640          2,660

ctaaataataaatagtctaagtatgttcaatgagaacaaaaaagctctatctatatttttctatcctatttcatttatatccttttcattttgaactcaccagcttc
gatttattatttatcagattcatacaagttactcttgttttttcgagatagatataaaaagataggataaagtaaatataggaaaagtaaaacttgagtggtcgaag
                                        F25B3.3 promoter
  2,680          2,700          2,720          2,740          2,760          2,780

tcttcttcttcttgtccatatacttcttaaaagtctcgaacttctctctctcactctccatcaatttctttatggctaccgcttgcgctctgccgcttccgaagaaa
agaagaagaagaacaggtatatgaagaattttcagagcttgaagagagagagtgagaggtagttaaagaaataccgatggcgaacgcgagacggcgaaggcttcttt
                                        F25B3.3 promoter
            2,800          2,820          2,840          2,860          2,880

              BanII
              SacI                    BspDI
            Eco53kI                   ClaI
gaaggcgttggcagagctctcaattctatttttttctcgccgtaggctatcgatttctcagcttctcttctctcttcctcactccaccaccaccacttcgggcttctt
cttccgcaaccgtctcgagagttaagataaaaaaagagcggcatccgatagctaaagagtcgaagagaagagagaaggagtgaggtggtggtggtgaagcccgaagaa
                                        F25B3.3 promoter
       2,900          2,920          2,940          2,960          2,980

                                                                BcgI                      BssHII
cttttcttcattttcgtcttcttcttcatcctcttcttttttttttcagttccccctcactcctccccttatatgcgcgtgcgagaggggtgcaaaagcagcgcgcat
gaaaagaagtaaaagcagaagaagaagtaggagaagaaaaaaaaagtcaagggggagtgaggaggggaatatacgcgcacgctctccccacgttttcgtcgcgcgta
                                        F25B3.3 promoter
 3,000          3,020          3,040          3,060          3,080          3,100
```

```
ccgagaattgaaacgagaaaacggagacgcagcagcagttcgtcctcagaaaaatagccagtaaaaagagaaaaagatagagagagacctatcgcattttattttca
ggctcttaactttgctcttttgcctctgcgtcgtcgtcaagcaggagtctttttatcggtcatttttctctttttctatctctctctggatagcgtaaaataaaagt
```

F25B3.3 promoter

3,120 3,140 3,160 3,180 3,200

BstAPI BglII

```
aattgcaattcctgcaattcatgtgtgcgtgtgcccattttcaattcttcccgttatttttcagaccattaccaacgttctttctagatcttgagacaatcttcctt
ttaacgttaaggacgttaagtacacacgcacacgggtaaaagttaagaagggcaataaaaagtctggtaatggttgcaagaaagatctagaactctgttagaaggaa
```

F25B3.3 promoter

3,220 3,240 3,260 3,280 3,300

TspMI
BsoBI
XmaI
AvaI
BamHI SmaI MscI PpuMI

```
ctgctcaatcgttcgtcgtgaagacggcatcgacgacgacgacgatCagcagtaaagttgaGGATCCCCGGGATTGGCCAAAGGACCCAAAGGTATGTTTCGAATGA
gacgagttagcaagcagcacttctgccgtagctgctgctgctgctaGtcgtcatttcaactCCTAGGGGCCCTAACCGGTTTCCTGGGTTTCCATACAAAGCTTACT
```

F25B3.3 promoter SynIVS.A

3,320 3,340 3,360 3,380 3,400 3,420

HincII
AccI NcoI
SgrDI KpnI
BmtI BtgI
PpuMI NheI SalI Acc65I BaeI

```
TACTAACATAACATAGAACATTTTCAGGAGGACCCTTGGCTAGCGTCGACGGTACCATGGTCTCAAAGGGTGAAGAAGATAACATGGCAATTATTAAAGAGTTTATG
ATGATTGTATTGTATCTTGTAAAAGTCCTCCTGGGAACCGATCGCAGCTGCCATGGTACCAGAGTTTCCCACTTCTTCTATTGTACCGTTAATAATTTCTCAAATAC
```

SynIVS.A rf21.28 mCherry with intron

3,440 3,460 3,480 3,500 3,520

```
CGTTTCAAGGTGCATATGGAGGGATCTGTCAATGGGCATGAGTTTGAAATTGAAGGTGAAGGAGAAGGCCGACCATATGAGGGAACACAAACCGCAAAACTAAAGgt
GCAAAGTTCCACGTATACCTCCCTAGACAGTTACCCGTACTCAAACTTTAACTTCCACTTCCTCTTCCGGCTGGTATACTCCCTTGTGTTTGGCGTTTTGATTTCca
```

mCherry with intron

3,540 3,560 3,580 3,600 3,620

BstXI

```
aagtttaaacatatatatactaactaaccctgattatttaaattttcagGTAACTAAAGGCGGACCATTACCATTCGCCTGGGACATCCTCTCTCCACAGTTCATGT
ttcaaatttgtatatatatgattgattgggactaataaatttaaaagtcCATTGATTTCCGCCTGGTAATGGTAAGCGGACCCTGTAGGAGAGAGGTGTCAAGTACA
```

mCherry with intron

3,640 3,660 3,680 3,700 3,720 3,740

MluI

ATGGAAGTAAAGCTTATGTTAAACATCCGGCAGATATACCAGATTATTTGAAACTTTCATTCCCGGAGGGTTTTAAGTGGGAACGCGTAATGAATTTTGAAGACGGA
TACCTTCATTTCGAATACAATTTGTAGGCCGTCTATATGGTCTAATAAACTTTGAAAGTAAGGGCCTCCCAAAATTCACCCTTGCGCATTACTTAAAACTTCTGCCT

mCherry with intron

3,760 3,780 3,800 3,820 3,840

GGAGTTGTTACAGTGACGCAAGACTCAAGgtaagtttaaacagttcggtactaactaaccatacatatttaaattttcagCCTCCAAGATGGAGAATTTATTTATAA
CCTCAACAATGTCACTGCGTTCTGAGTTCcattcaaatttgtcaagccatgattgattggtatgtataaatttaaaagtcGGAGGTTCTACCTCTTAAATAAATATT

mCherry with intron

3,860 3,880 3,900 3,920 3,940

AGTCAAACTTCGAGGAACGAATTTCCCCTCGGATGGACCTGTTATGCAGAAGAAGACTATGGGATGGGAAGCTTCAAGTGAAAGAATGTACCCTGAAGACGGTGCTC
TCAGTTTGAAGCTCCTTGCTTAAAGGGGAGCCTACCTGGACAATACGTCTTCTTCTGATACCCTACCCTTCGAAGTTCACTTTCTTACATGGGACTTCTGCCACGAG

mCherry with intron

3,960 3,980 4,000 4,020 4,040 4,060

AflII BbvCI

TTAAGGGAGAGATTAAACAACGTCTTAAATTGAAAGATGGAGGACATTACGATGCTGAGgtaagtttaaacatgattttactaactaactaatctgatttaaatttt
AATTCCCTCTCTAATTTGTTGCAGAATTTAACTTTCTACCTCCTGTAATGCTACGACTCcattcaaatttgtactaaaatgattgattgattagactaaatttaaaa

mCherry with intron

4,080 4,100 4,120 4,140 4,160

PvuII EcoRV

cagGTGAAGACAACTTACAAAGCCAAAAAACCAGTTCAGCTGCCAGGAGCGTACAATGTTAATATTAAACTGGATATCACCTCCCACAACGAGGATTACACTATCGT
gtcCACTTCTGTTGAATGTTTCGGTTTTTTGGTCAAGTCGACGGTCCTCGCATGTTACAATTATAATTTGACCTATAGTGGAGGGTGTTGCTCCTAATGTGATAGCA

mCherry with intron

4,180 4,200 4,220 4,240 4,260 4,280

EagI BsaBI

TGAGCAATATGAAAGAGCTGAAGGGCGGCACTCGACAGGTGGCATGGATGAATTGTATAAGTAGCGCATCGGCCGCTGTCATCAGATCGCCATCTCGCGCCCGTGCC
ACTCGTTATACTTTCTCGACTTCCCGCCGTGAGCTGTCCACCGTACCTACTTAACATATTCATCGCGTAGCCGGCGACAGTAGTCTAGCGGTAGAGCGCGGGCACGG

mCherry with intron

4,300 4,320 4,340 4,360 4,380

TCTGACTTCTAAGTCCAATTACTCTTCAACATCCCTACATGCTCTTTCTCCCTGTGCTCCCACCCCCTATTTTTGTTATTATCAAAAAACTTCTCTTAATTTCTTTG
AGACTGAAGATTCAGGTTAATGAGAAGTTGTAGGGATGTACGAGAAAGAGGGACACGAGGGTGGGGGATAAAAACAATAATAGTTTTTTGAAGAGAATTAAAGAAAC

unc-54 3'UTR

4,400 4,420 4,440 4,460 4,480

TTTTTTAGCTTCTTTTAAGTCACCTCTAACAATGAAATTGTGTAGATTCAAAAATAGAATTAATTCGTAATAAAAAGTCGAAAAAAATTGTGCTCCCTCCCCCCATT
AAAAAATCGAAGAAAATTCAGTGGAGATTGTTACTTTAACACATCTAAGTTTTTATCTTAATTAAGCATTATTTTTCAGCTTTTTTTAACACGAGGGAGGGGGGTAA

unc-54 3'UTR

4,500 4,520 4,540 4,560 4,580 4,600

BsrGI

AATAATAATTCTATCCCAAAATCTACACAATGTTCTGTGTACACTTCTTATGTTTTTTACTTCTGATAAATTTTTTTGAAACATCATAGAAAAAACCGCACACAAAA
TTATTATTAAGATAGGGTTTTAGATGTGTTACAAGACACATGTGAAGAATACAAAAAATGAAGACTATTTAAAAAAACTTTGTAGTATCTTTTTTGGCGTGTGTTTT

unc-54 3'UTR

4,620 4,640 4,660 4,680 4,700

BmgBI

TACCTTATCATATGTTACGTTTCAGTTTATGACCGCAATTTTTATTTCTTCGCACGTCTGGGCCTCTCATGACGTCAAATCATGCTCATCGTGAAAAAGTTTTGGAG
ATGGAATAGTATACAATGCAAAGTCAAATACTGGCGTTAAAAATAAAGAAGCGTGCAGACCCGGAGAGTACTGCAGTTTAGTACGAGTAGCACTTTTTCAAAACCTC

unc-54 3'UTR

4,720 4,740 4,760 4,780 4,800

TATTTTTGGAATTTTTCAATCAAGTGAAAGTTTATGAAATTAATTTTCCTGCTTTTGCTTTTTGGGGTTTCCCCTATTGTTTGTCAAGATTTCGAGGACGGCGTTTT
ATAAAAACCTTAAAAAGTTAGTTCACTTTCAAATACTTTAATTAAAAGGACGAAAACGAAAAACCCCAAAGGGGATAACAAACAGTTCTAAAGCTCCTGCCGCAAAA

unc-54 3'UTR

4,820 4,840 4,860 4,880 4,900 4,920

TCTTGCTAAAATCACAAGTATTGATGAGCACGATGCAAGAAAGATCGGAAGAAGGTTTGGGTTTGAGGCTCAGTGGAAGGTGAGTAGAAGTTGATAATTTGAAAGTG
AGAACGATTTTAGTGTTCATAACTACTCGTGCTACGTTCTTTCTAGCCTTCTTCCAAACCCAAACTCCGAGTCACCTTCCACTCATCTTCAACTATTAAACTTTCAC

unc-54 3'UTR

4,940 4,960 4,980 5,000 5,020

BanII
PspOMI
BsiWI ApaI
SpeI EagI SfiI

GAGTAGTGTCTATGGGGTTTTTGCCTTAAATGACAGAATACATTCCCAATATACCAAACATAACTGTTTCCTACTAGTCGGCCGTACGGGCCCTTTCGTCTCGCGCG
CTCATCACAGATACCCCAAAAACGGAATTTACTGTCTTATGTAAGGGTTATATGGTTTGTATTGACAAAGGATGATCAGCCGGCATGCCCGGGAAAGCAGAGCGCGC

unc-54 3'UTR

5,040 5,060 5,080 5,100 5,120

TTTCGGTGATGACGGTGAAAACCTCTGACACATGCAGCTCCCGGAGACGGTCACAGCTTGTCTGTAAGCGGATGCCGGGAGCAGACAAGCCCGTCAGGGCGCGTCAG
AAAGCCACTACTGCCACTTTTGGAGACTGTGTACGTCGAGGGCCTCTGCCAGTGTCGAACAGACATTCGCCTACGGCCCTCGTCTGTTCGGGCAGTCCCGCGCAGTC

5,140 5,160 5,180 5,200 5,220 5,240

BstAPI

CGGGTGTTGGCGGGTGTCGGGGCTGGCTTAACTATGCGGCATCAGAGCAGATTGTACTGAGAGTGCACCATATGCGGTGTGAAATACCGCACAGATGCGTAAGGAGA
GCCCACAACCGCCCACAGCCCCGACCGAATTGATACGCCGTAGTCTCGTCTAACATGACTCTCACGTGGTATACGCCACACTTTATGGCGTGTCTACGCATTCCTCT

5,260 5,280 5,300 5,320 5,340

SfiI
AflII

AAATACCGCATCAGGCGGCCTTAAGGGCCTCGTGATACGCCTATTTTTATAGGTTAATGTCATGATAATAATGGTTTCTTAGACGTCAGGTGGCACTTTTCGGGGAA
TTTATGGCGTAGTCCGCCGGAATTCCCGGAGCACTATGCGGATAAAAATATCCAATTACAGTACTATTATTACCAAAGAATCTGCAGTCCACCGTGAAAAGCCCCTT

5,360 5,380 5,400 5,420 5,440

BciVI

ATGTGCGCGGAACCCCTATTTGTTTATTTTTCTAAATACATTCAAATATGTATCCGCTCATGAGACAATAACCCTGATAAATGCTTCAATAATATTGAAAAAGGAAG
TACACGCGCCTTGGGGATAAACAAATAAAAAGATTTATGTAAGTTTATACATAGGCGAGTACTCTGTTATTGGGACTATTTACGAAGTTATTATAACTTTTTCCTTC

AmpR promoter

5,460 5,480 5,500 5,520 5,540 5,560

AGTATGAGTATTCAACATTTCCGTGTCGCCCTTATTCCCTTTTTTGCGGCATTTTGCCTTCCTGTTTTTGCTCACCCAGAAACGCTGGTGAAAGTAAAAGATGCTGA
TCATACTCATAAGTTGTAAAGGCACAGCGGGAATAAGGGAAAAAACGCCGTAAAACGGAAGGACAAAAACGAGTGGGTCTTTGCGACCACTTTCATTTTCTACGACT

AmpR

5,580 5,600 5,620 5,640 5,660

AGATCAGTTGGGTGCACGAGTGGGTTACATCGAACTGGATCTCAACAGCGGTAAGATCCTTGAGAGTTTTCGCCCCGAAGAACGTTTTCCAATGATGAGCACTTTTA
TCTAGTCAACCCACGTGCTCACCCAATGTAGCTTGACCTAGAGTTGTCGCCATTCTAGGAACTCTCAAAAGCGGGGCTTCTTGCAAAAGGTTACTACTCGTGAAAAT

AmpR

5,680 5,700 5,720 5,740 5,760

BcgI ScaI

AAGTTCTGCTATGTGGCGCGGTATTATCCCGTATTGACGCCGGGCAAGAGCAACTCGGTCGCCGCATACACTATTCTCAGAATGACTTGGTTGAGTACTCACCAGTC
TTCAAGACGATACACCGCGCCATAATAGGGCATAACTGCGGCCCGTTCTCGTTGAGCCAGCGGCGTATGTGATAAGAGTCTTACTGAACCAACTCATGAGTGGTCAG

AmpR

5,780 5,800 5,820 5,840 5,860 5,880

PvuI

ACAGAAAAGCATCTTACGGATGGCATGACAGTAAGAGAATTATGCAGTGCTGCCATAACCATGAGTGATAACACTGCGGCCAACTTACTTCTGACAACGATCGGAGG
TGTCTTTTCGTAGAATGCCTACCGTACTGTCATTCTCTTAATACGTCACGACGGTATTGGTACTCACTATTGTGACGCCGGTTGAATGAAGACTGTTGCTAGCCTCC

AmpR

5,900 5,920 5,940 5,960 5,980

ACCGAAGGAGCTAACCGCTTTTTTGCACAACATGGGGGATCATGTAACTCGCCTTGATCGTTGGGAACCGGAGCTGAATGAAGCCATACCAAACGACGAGCGTGACA
TGGCTTCCTCGATTGGCGAAAAAACGTGTTGTACCCCCTAGTACATTGAGCGGAACTAGCAACCCTTGGCCTCGACTTACTTCGGTATGGTTTGCTGCTCGCACTGT

AmpR

6,000 6,020 6,040 6,060 6,080

FspI

CCACGATGCCTGTAGCAATGGCAACAACGTTGCGCAAACTATTAACTGGCGAACTACTTACTCTAGCTTCCCGGCAACAATTAATAGACTGGATGGAGGCGGATAAA
GGTGCTACGGACATCGTTACCGTTGTTGCAACGCGTTTGATAATTGACCGCTTGATGAATGAGATCGAAGGGCCGTTGTTAATTATCTGACCTACCTCCGCCTATTT

AmpR

6,100 6,120 6,140 6,160 6,180 6,200

NmeAIII BsrFI BpmI

GTTGCAGGACCACTTCTGCGCTCGGCCCTTCCGGCTGGCTGGTTTATTGCTGATAAATCTGGAGCCGGTGAGCGTGGGTCTCGCGGTATCATTGCAGCACTGGGGCC
CAACGTCCTGGTGAAGACGCGAGCCGGGAAGGCCGACCGACCAAATAACGACTATTTAGACCTCGGCCACTCGCACCCAGAGCGCCATAGTAACGTCGTGACCCCGG

AmpR

6,220 6,240 6,260 6,280 6,300

BmrI AhdI

AGATGGTAAGCCCTCCCGTATCGTAGTTATCTACACGACGGGGAGTCAGGCAACTATGGATGAACGAAATAGACAGATCGCTGAGATAGGTGCCTCACTGATTAAGC
TCTACCATTCGGGAGGGCATAGCATCAATAGATGTGCTGCCCCTCAGTCCGTTGATACCTACTTGCTTTATCTGTCTAGCGACTCTATCCACGGAGTGACTAATTCG

AmpR

6,320 6,340 6,360 6,380 6,400 6,420

ATTGGTAACTGTCAGACCAAGTTTACTCATATATACTTTAGATTGATTTAAAACTTCATTTTTAATTTAAAAGGATCTAGGTGAAGATCCTTTTTGATAATCTCATG
TAACCATTGACAGTCTGGTTCAAATGAGTATATATGAAATCTAACTAAATTTTGAAGTAAAAATTAAATTTTCCTAGATCCACTTCTAGGAAAAACTATTAGAGTAC

6,440 6,460 6,480 6,500 6,520

ACCAAAATCCCTTAACGTGAGTTTTCGTTCCACTGAGCGTCAGACCCCGTAGAAAAGATCAAAGGATCTTCTTGAGATCCTTTTTTTCTGCGCGTAATCTGCTGCTT
TGGTTTTAGGGAATTGCACTCAAAAGCAAGGTGACTCGCAGTCTGGGGCATCTTTTCTAGTTTCCTAGAAGAACTCTAGGAAAAAAAGACGCGCATTAGACGACGAA

ColE1 origin

6,540 6,560 6,580 6,600 6,620

GCAAACAAAAAAACCACCGCTACCAGCGGTGGTTTGTTTGCCGGATCAAGAGCTACCAACTCTTTTTCCGAAGGTAACTGGCTTCAGCAGAGCGCAGATACCAAATA
CGTTTGTTTTTTTGGTGGCGATGGTCGCCACCAAACAAACGGCCTAGTTCTCGATGGTTGAGAAAAAGGCTTCCATTGACCGAAGTCGTCTCGCGTCTATGGTTTAT

ColE1 origin

6,640 6,660 6,680 6,700 6,720 6,740

AlwNI

CTGTCCTTCTAGTGTAGCCGTAGTTAGGCCACCACTTCAAGAACTCTGTAGCACCGCCTACATACCTCGCTCTGCTAATCCTGTTACCAGTGGCTGCTGCCAGTGGC
GACAGGAAGATCACATCGGCATCAATCCGGTGGTGAAGTTCTTGAGACATCGTGGCGGATGTATGGAGCGAGACGATTAGGACAATGGTCACCGACGACGGTCACCG

ColE1 origin

6,760 6,780 6,800 6,820 6,840

BseYI

GATAAGTCGTGTCTTACCGGGTTGGACTCAAGACGATAGTTACCGGATAAGGCGCAGCGGTCGGGCTGAACGGGGGGTTCGTGCACACAGCCCAGCTTGGAGCGAAC
CTATTCAGCACAGAATGGCCCAACCTGAGTTCTGCTATCAATGGCCTATTCCGCGTCGCCAGCCCGACTTGCCCCCCAAGCACGTGTGTCGGGTCGAACCTCGCTTG

ColE1 origin

6,860 6,880 6,900 6,920 6,940

BciVI

GACCTACACCGAACTGAGATACCTACAGCGTGAGCATTGAGAAAGCGCCACGCTTCCCGAAGGGAGAAAGGCGGACAGGTATCCGGTAAGCGGCAGGGTCGGAACAG
CTGGATGTGGCTTGACTCTATGGATGTCGCACTCGTAACTCTTTCGCGGTGCGAAGGGCTTCCCTCTTTCCGCCTGTCCATAGGCCATTCGCCGTCCCAGCCTTGTC

ColE1 origin

6,960 6,980 7,000 7,020 7,040 7,060

GAGAGCGCACGAGGGAGCTTCCAGGGGGAAACGCCTGGTATCTTTATAGTCCTGTCGGGTTTCGCCACCTCTGACTTGAGCGTCGATTTTTGTGATGCTCGTCAGGG
CTCTCGCGTGCTCCCTCGAAGGTCCCCCTTTGCGGACCATAGAAATATCAGGACAGCCCAAAGCGGTGGAGACTGAACTCGCAGCTAAAAACACTACGAGCAGTCCC

ColE1 origin

7,080 7,100 7,120 7,140 7,160

PciI

GGGCGGAGCCTATGGAAAAACGCCAGCAACGCGGCCTTTTTACGGTTCCTGGCCTTTTGCTGGCCTTTTGCTCACATGTTCTTTCCTGCGTTATCCCCTGATTCTGT
CCCGCCTCGGATACCTTTTTGCGGTCGTTGCGCCGGAAAAATGCCAAGGACCGGAAAACGACCGGAAAACGAGTGTACAAGAAAGGACGCAATAGGGGACTAAGACA

ColE1 origin

7,180 7,200 7,220 7,240 7,260

BspQI
SapI

GGATAACCGTATTACCGCCTTTGAGTGAGCTGATACCGCTCGCCGCAGCCGAACGACCGAGCGCAGCGAGTCAGTGAGCGAGGAAGCGGAAGAGCGCCCAATACGCA
CCTATTGGCATAATGGCGGAAACTCACTCGACTATGGCGAGCGGCGTCGGCTTGCTGGCTCGCGTCGCTCAGTCACTCGCTCCTTCGCCTTCTCGCGGGTTATGCGT

7,280 7,300 7,320 7,340 7,360 7,380

PvuII

AACCGCCTCTCCCCGCGCGTTGGCCGATTCATTAATGCAGCTGGCACGACAGGTTTCCCGACTGGAAAGCGGGCAGTGAGCGCAACGCAATTAATGTGAGTTAGCTC
TTGGCGGAGAGGGGCGCGCAACCGGCTAAGTAATTACGTCGACCGTGCTGTCCAAAGGGCTGACCTTTCGCCCGTCACTCGCGTTGCGTTAATTACACTCAATCGAG

7,400 7,420 7,440 7,460 7,480

ACTCATTAGGCACCCCAGGCTTTACACTTTATGCTTCCGGCTCGTATGTTGTGTGGAATTGTGAGCGGATAACAATTTCACACAGGAAACAGCT
TGAGTAATCCGTGGGGTCCGAAATGTGAAATACGAAGGCCGAGCATACAACACACCTTAACACTCGCCTATTGTTAAAGTGTGTCCTTTGTCGA

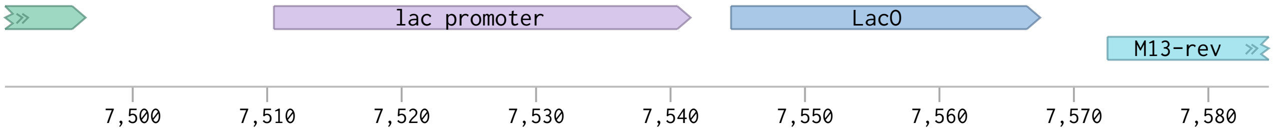

2017/10/10 15:39:10

pQUAS_ChR2_GFP (5612 bp)

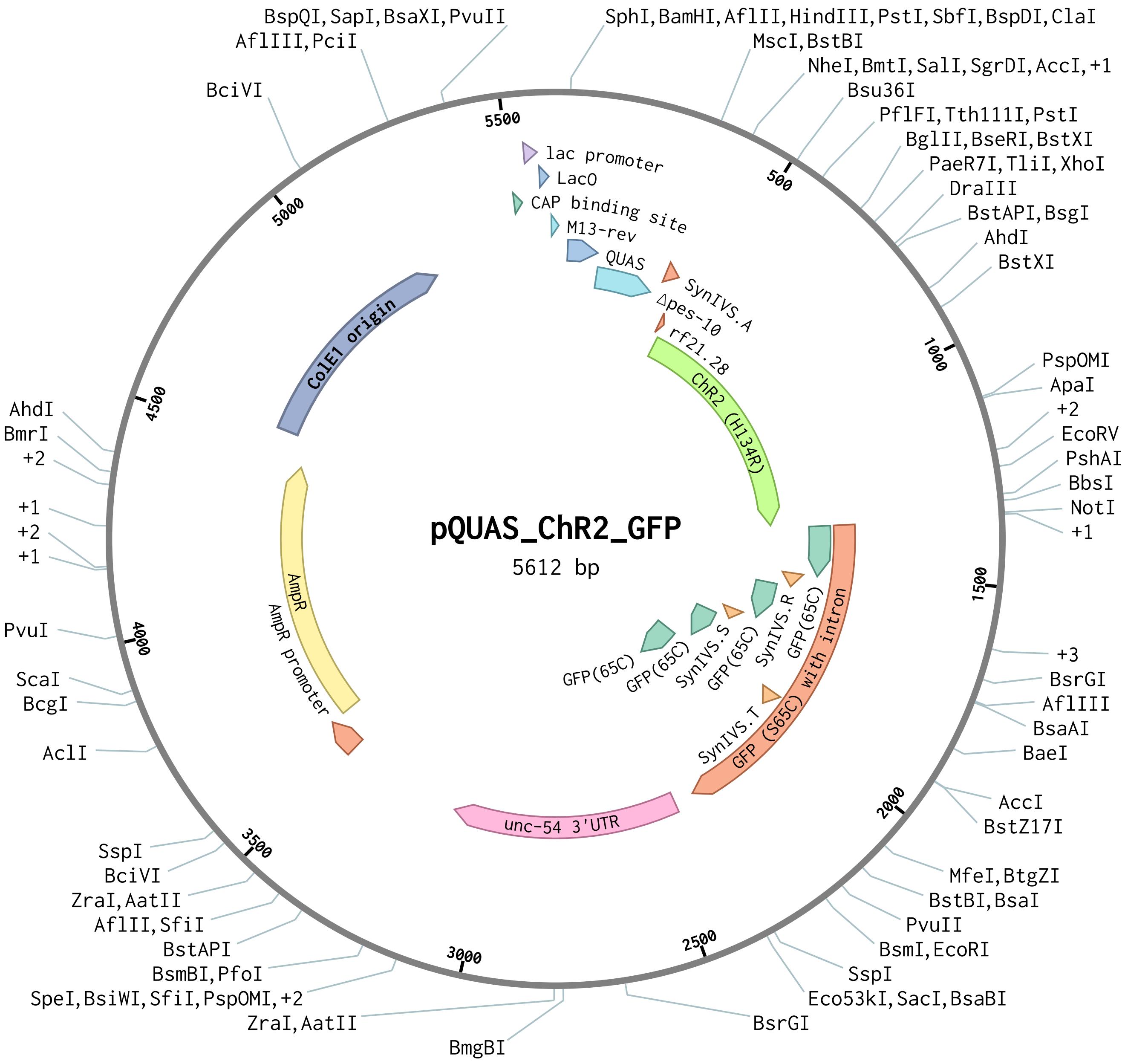

pQUAS_ChR2_GFP (5612 bp)

BamHI
SphI

ATGACCATGATTACGCCACTTACTTGCATGCGGATCCGGGTAATCGCTTATCCtcGGATAAACAATTATCCtcacGGGTAATCGCTTATCCgctcGGGTAATCGCTT
TACTGGTACTAATGCGGTGAATGAACGTACGCCTAGGCCCATTAGCGAATAGGagCCTATTTGTTAATAGGagtgCCCATTAGCGAATAGGcgagCCCATTAGCGAA

QUAS

20 40 60 80 100

BspDI
ClaI
HindIII SbfI
AflII PstI

ATCCtcGGGTAATCGCTTATCCttAAGCTTCCTGCAGGATCGATTTTTTGCAAATTACGAGCGTTGTAGGGGGCGGAgCGATAGGTCCTATAGGTTTTgGTATATCA
TAGGagCCCATTAGCGAATAGGaaTTCGAAGGACGTCCTAGCTAAAAAACGTTTAATGCTCGCAACATCCCCCGCCTcGCTATCCAGGATATCCAAAAcCATATAGT

QUAS Δpes-10

120 140 160 180 200

TCATTCATTCATTCATTGGTACATTCATTTACCCACCTTCCTCTTTCTGAGCTTCTCTGGAGTTCTGTGCTTCCTTTTTCCCTTATCTTTATACTGTAATTTTTAAC
AGTAAGTAAGTAAGTAACCATGTAAGTAAATGGGTGGAAGGAGAAAGACTCGAAGAGACCTCAAGACACGAAGGAAAAAGGGAATAGAAATATGACATTAAAAATTG

Δpes-10

220 240 260 280 300 320

HincII
AccI
SgrDI
BmtI
MscI BstBI NheI SalI

TTTCAGCCGGGATTGGCCAAAGGACCCAAAGGTATGTTTCGAATGATACTAACATAACATAGAACATTTTCAGGAGGACCCTTGGCTAGCGTCGACGatggactatg
AAAGTCGGCCCTAACCGGTTTCCTGGGTTTCCATACAAAGCTTACTATGATTGTATTGTATCTTGTAAAAGTCCTCCTGGGAACCGATCGCAGCTGCtacctgatac

SynIVS.A rf21.28

340 360 380 400 420

Bsu36I

gcggcgctttgtctgccgtcggacgcgaacttttgttcgttactaatcctgtggtggtgaacgggtccgtcctggtccctgaggatcaatgttactgtgccggatgg
cgccgcgaaacagacggcagcctgcgcttgaaaacaagcaatgattaggacaccaccacttgcccaggcaggaccagggactcctagttacaatgacacggcctacc

ChR2 (H134R)

440 460 480 500 520

Tth111I
PflFI
PstI

attgaatctcgcggcacgaacggcgctcagaccgcgtcaaatgtcctgcagtggcttgcagcaggattcagcattttgctgctgatgttctatgcctaccaaacctg
taacttagagcgccgtgcttgccgcgagtctggcgcagtttacaggacgtcaccgaacgtcgtcctaagtcgtaaaacgacgactacaagatacggatggtttggac

ChR2 (H134R)

540 560 580 600 620 640

PaeR7I
XhoI
BglII BseRI BstXI TliI

gaaatctacatgcggctgggaggagatctatgtgtgcgccattgaaatggttaaggtgattctcgagttctttttgagtttaagaatccctctatgctctaccttg
ctttagatgtacgccgaccctcctctagatacacacgcggtaactttaccaattccactaagagctcaagaaaaactcaaattcttagggagatacgagatggaac

ChR2 (H134R)

660 680 700 720 740

DraIII BstAPI BsgI

ccacaggacaccgggtgcagtggctgcgctatgcagagtggctgctcacttgtcctgtcatccttatccgcctgagcaacctcaccggcctgagcaacgactacagc
ggtgtcctgtggcccacgtcaccgacgcgatacgtctcaccgacgagtgaacaggacagtaggaataggcggactcgttggagtggccggactcgttgctgatgtcg

ChR2 (H134R)

760 780 800 820 840

AhdI BstXI

aggagaaccatgggactccttgtctcagacatcgggactatcgtgtgggggctaccagcgccatggcaaccggctatgttaaagtcatcttcttttgtcttggatt
tcctcttggtaccctgaggaacagagtctgtagccctgatagcacacccccgatggtcgcggtaccgttggccgatacaatttcagtagaagaaaacagaacctaa

ChR2 (H134R)

860 880 900 920 940 960

gtgctatggcgcgaacacatttttcacgccgccaaagcatatatcgagggttatcatactgtgccaaagggtcggtgccgccaggtcgtgaccggcatggcatggc
cacgataccgcgcttgtgtaaaaagtgcggcggtttcgtatatagctcccaatagtatgacacggtttcccagccacggcggtccagcactggccgtaccgtaccg

ChR2 (H134R)

980 1,000 1,020 1,040 1,060

ApaI
PspOMI

tgtttttcgtgagctggggtatgttcccaattctcttcattttggggcccgaaggttttggcgtcctgagcgtctatggctccaccgtaggtcacacgattattgat
acaaaaagcactcgaccccatacaagggttaagagaagtaaaaccccgggcttccaaaaccgcaggactcgcagataccgaggtggcatccagtgtgctaataacta

ChR2 (H134R)

1,080 1,100 1,120 1,140 1,160

BspMI
BfuAI
EcoRV

ctgatgagtaaaaattgttgggggttgttgggacactacctgcgcgtcctgatccacgagcacatattgattcacggagatatccgcaaaaccaccaaactgaacat
gactactcatttttaacaacccccaacaaccctgtgatggacgcgcaggactaggtgctcgtgtataactaagtgcctctataggcgttttggtggtttgacttgta

ChR2 (H134R)

1,180 1,200 1,220 1,240 1,260 1,280

NmeAIII
PshAI BbsI NotI

cggcggaacggagatcgaggtcgagactctcgtcgaagacgaagccgaggccggagccgtgccagcggccgccaccATGAGTAAAGGAGAAGAACTTTTCACTGGAG
gccgccttgcctctagctccagctctgagagcagcttctgcttcggctccggcctcggcacggtcgccggcggtggTACTCATTTCCTCTTCTTGAAAAGTGACCTC

GFP(65C)
ChR2 (H134R)
GFP (S65C) with intron

1,300 1,320 1,340 1,360 1,380

TTGTCCCAATTCTTGTTGAATTAGATGGTGATGTTAATGGGCACAAATTTTCTGTCAGTGGAGAGGGTGAAGGTGATGCAACATACGGAAAACTTACCCTTAAATTT
AACAGGGTTAAGAACAACTTAATCTACCACTACAATTACCCGTGTTTAAAAGACAGTCACCTCTCCCACTTCCACTACGTTGTATGCCTTTTGAATGGGAATTTAAA

GFP(65C)
GFP (S65C) with intron

1,400 1,420 1,440 1,460 1,480

ATTTGCACTACTGGAAAACTACCTGTTCCATGGgtaagtttaaacatatatatactaactaaccctgattatttaaattttcagCCAACACTTGTCACTACTTTCTg
TAAACGTGATGACCTTTTGATGGACAAGGTACCcattcaaatttgtatatatatgattgattgggactaataaatttaaaagtcGGTTGTGAACAGTGATGAAAGAc

GFP(65C) SynIVS.R GFP(65C)
GFP (S65C) with intron

1,500 1,520 1,540 1,560 1,580 1,600

PaeR7I
XhoI
TliI
BsrGI

TTATGGTGTTCAATGCTTcTCgAGATACCCAGATCATATGAAACgGCATGACTTTTTCAAGAGTGCCATGCCCGAAGGTTATGTACAGGAAAGAACTATATTTTTCA
AATACCACAAGTTACGAAgAGcTCTATGGGTCTAGTATACTTTGcCGTACTGAAAAAGTTCTCACGGTACGGGCTTCCAATACATGTCCTTTCTTGATATAAAAAGT

GFP(65C)
GFP (S65C) with intron

1,620 1,640 1,660 1,680 1,700

BsaAI
AflIII

AAGATGACGGGAACTACAAGACACgtaagtttaaacagttcggtactaactaaccatacatatttaaattttcagGTGCTGAAGTCAAGTTTGAAGGTGATACCCTT
TTCTACTGCCCTTGATGTTCTGTGcattcaaatttgtcaagccatgattgattggtatgtataaatttaaaagtcCACGACTTCAGTTCAAACTTCCACTATGGGAA

GFP(65C) SynIVS.S GFP(65C)
GFP (S65C) with intron

1,720 1,740 1,760 1,780 1,800

```
BaeI  AccI  BstZ17I
GTTAATAGAATCGAGTTAAAAGGTATTGATTTTAAAGAAGATGGAAACATTCTTGGACACAAATTGGAATACAACTATAACTCACACAATGTATACATCATGGCAGA
CAATTATCTTAGCTCAATTTTCCATAACTAAAATTTCTTCTACCTTTGTAAGAACCTGTGTTTAACCTTATGTTGATATTGAGTGTGTTACATATGTAGTACCGTCT
GFP(65C)
GFP (S65C) with intron
1,820  1,840  1,860  1,880  1,900  1,920

CAAACAAAAGAATGGAATCAAAGTTgtaagtttaaacatgattttactaactaactaatctgatttaaattttcagAACTTCAAAATTAGACACAACATTGAAGATG
GTTTGTTTTCTTACCTTAGTTTCAAcattcaaatttgtactaaaatgattgattgattagactaaatttaaaagtcTTGAAGTTTTAATCTGTGTTGTAACTTCTAC
GFP(65C)  SynIVS.T  GFP(65C)
GFP (S65C) with intron
1,940  1,960  1,980  2,000  2,020

MfeI  BtgZI  BstBI
GAAGCGTTCAACTAGCAGACCATTATCAACAAAATACTCCAATTGGCGATGGCCCTGTCCTTTTACCAGACAACCATTACCTGTCCACACAATCTGCCCTTTCGAAA
CTTCGCAAGTTGATCGTCTGGTAATAGTTGTTTTATGAGGTTAACCGCTACCGGGACAGGAAAATGGTCTGTTGGTAATGGACAGGTGTGTTAGACGGGAAAGCTTT
GFP(65C)
GFP (S65C) with intron
2,040  2,060  2,080  2,100  2,120  2,140

BsaI  PvuII  BsmI  EcoRI
GATCCCAACGAAAAGAGAGACCACATGGTCCTTCTTGAGTTTGTAACAGCTGCTGGGATTACACATGGCATGGATGAACTATACAAATAGCATTCGTAGAATTCCAA
CTAGGGTTGCTTTTCTCTCTGGTGTACCAGGAAGAACTCAAACATTGTCGACGACCCTAATGTGTACCGTACCTACTTGATATGTTTATCGTAAGCATCTTAAGGTT
GFP(65C)
GFP (S65C) with intron
2,160  2,180  2,200  2,220  2,240

SspI
CTGAGCGCCGGTCGCTACCATTACCAACTTGTCTGGTGTCAAAAATAATAGGGGCCGCTGTCATCAGAgtaagtttaaactgagttctactaactaacgagtaatat
GACTCGCGGCCAGCGATGGTAATGGTTGAACAGACCACAGTTTTTATTATCCCCGGCGACAGTAGTCTcattcaaatttgactcaagatgattgattgctcattata
GFP (S65C) with intron
2,260  2,280  2,300  2,320  2,340

SacI  Eco53kI  BsaBI
ttaaattttcagGAGCTCCGCATCGGCCGCTGTCATCAGATCGCCATCTCGCGCCCGTGCCTCTGACTTCTAAGTCCAATTACTCTTCAACATCCCTACATGCTCTT
aatttaaaagtcCTCGAGGCGTAGCCGGCGACAGTAGTCTAGCGGTAGAGCGCGGGCACGGAGACTGAAGATTCAGGTTAATGAGAAGTTGTAGGGATGTACGAGAA
unc-54 3'UTR
2,360  2,380  2,400  2,420  2,440  2,460
```

TCTCCCTGTGCTCCCACCCCCTATTTTTGTTATTATCAAAAAACTTCTCTTAATTTCTTTGTTTTTTAGCTTCTTTTAAGTCACCTCTAACAATGAAATTGTGTAGA
AGAGGGACACGAGGGTGGGGGATAAAAACAATAATAGTTTTTTGAAGAGAATTAAAGAAACAAAAAATCGAAGAAAATTCAGTGGAGATTGTTACTTTAACACATCT

unc-54 3'UTR

2,480 2,500 2,520 2,540 2,560

BsrGI

TTCAAAAATAGAATTAATTCGTAATAAAAAGTCGAAAAAAATTGTGCTCCCTCCCCCCATTAATAATAATTCTATCCCAAAATCTACACAATGTTCTGTGTACACTT
AAGTTTTTATCTTAATTAAGCATTATTTTTCAGCTTTTTTTAACACGAGGGAGGGGGGTAATTATTATTAAGATAGGGTTTTAGATGTGTTACAAGACACATGTGAA

unc-54 3'UTR

2,580 2,600 2,620 2,640 2,660

CTTATGTTTTTTACTTCTGATAAATTTTTTTGAAACATCATAGAAAAAACCGCACACAAAATACCTTATCATATGTTACGTTTCAGTTTATGACCGCAATTTTTATT
GAATACAAAAAATGAAGACTATTTAAAAAAACTTTGTAGTATCTTTTTTGGCGTGTGTTTTATGGAATAGTATACAATGCAAAGTCAAATACTGGCGTTAAAAATAA

unc-54 3'UTR

2,680 2,700 2,720 2,740 2,760 2,780

AatII
BmgBI ZraI

TCTTCGCACGTCTGGGCCTCTCATGACGTCAAATCATGCTCATCGTGAAAAAGTTTTGGAGTATTTTTGGAATTTTTCAATCAAGTGAAAGTTTATGAAATTAATTT
AGAAGCGTGCAGACCCGGAGAGTACTGCAGTTTAGTACGAGTAGCACTTTTTCAAAACCTCATAAAAACCTTAAAAAGTTAGTTCACTTTCAAATACTTTAATTAAA

unc-54 3'UTR

2,800 2,820 2,840 2,860 2,880

TCCTGCTTTTGCTTTTTGGGGTTTCCCCTATTGTTTGTCAAGATTTCGAGGACGGCGTTTTTCTTGCTAAAATCACAAGTATTGATGAGCACGATGCAAGAAAGATC
AGGACGAAAACGAAAAACCCCAAAGGGGATAACAAACAGTTCTAAAGCTCCTGCCGCAAAAAGAACGATTTTAGTGTTCATAACTACTCGTGCTACGTTCTTTCTAG

unc-54 3'UTR

2,900 2,920 2,940 2,960 2,980

GGAAGAAGGTTTGGGTTTGAGGCTCAGTGGAAGGTGAGTAGAAGTTGATAATTTGAAAGTGGAGTAGTGTCTATGGGGTTTTTGCCTTAAATGACAGAATACATTCC
CCTTCTTCCAAACCCAAACTCCGAGTCACCTTCCACTCATCTTCAACTATTAAACTTTCACCTCATCACAGATACCCCAAAAACGGAATTTACTGTCTTATGTAAGG

unc-54 3'UTR

3,000 3,020 3,040 3,060 3,080 3,100

PspOMI
SfiI PfoI
SpeI BsiWI ApaI BsmBI BsmBI

CAATATACCAAACATAACTGTTTCCTACTAGTCGGCCGTACGGGCCCTTTCGTCTCGCGCGTTTCGGTGATGACGGTGAAAACCTCTGACACATGCAGCTCCCGGAG
GTTATATGGTTTGTATTGACAAAGGATGATCAGCCGGCATGCCCGGGAAAGCAGAGCGCGCAAAGCCACTACTGCCACTTTTGGAGACTGTGTACGTCGAGGGCCTC

unc-54 3'UTR

3,120 3,140 3,160 3,180 3,200

```
ACGGTCACAGCTTGTCTGTAAGCGGATGCCGGGAGCAGACAAGCCCGTCAGGGCGCGTCAGCGGGTGTTGGCGGGTGTCGGGGCTGGCTTAACTATGCGGCATCAGA
TGCCAGTGTCGAACAGACATTCGCCTACGGCCCTCGTCTGTTCGGGCAGTCCCGCGCAGTCGCCCACAACCGCCCACAGCCCCGACCGAATTGATACGCCGTAGTCT
        3,220               3,240               3,260               3,280               3,300

                    BstAPI                                                      AflII SfiI
GCAGATTGTACTGAGAGTGCACCATATGCGGTGTGAAATACCGCACAGATGCGTAAGGAGAAAATACCGCATCAGGCGGCCTTAAGGGCCTCGTGATACGCCTATTT
CGTCTAACATGACTCTCACGTGGTATACGCCACACTTTATGGCGTGTCTACGCATTCCTCTTTTATGGCGTAGTCCGCCGGAATTCCCGGAGCACTATGCGGATAAA
3,320               3,340               3,360               3,380               3,400               3,420

                                      ZraI AatII
TTATAGGTTAATGTCATGATAATAATGGTTTCTTAGACGTCAGGTGGCACTTTTCGGGGAAATGTGCGCGGAACCCCTATTTGTTTATTTTTCTAAATACATTCAAA
AATATCCAATTACAGTACTATTATTACCAAAGAATCTGCAGTCCACCGTGAAAAGCCCCTTTACACGCGCCTTGGGGATAAACAAATAAAAAGATTTATGTAAGTTT
                                                                  [AmpR promoter >>]
            3,440               3,460               3,480               3,500               3,520

               BciVI                           SspI
TATGTATCCGCTCATGAGACAATAACCCTGATAAATGCTTCAATAATATTGAAAAAGGAAGAGTATGAGTATTCAACATTTCCGTGTCGCCCTTATTCCCTTTTTTG
ATACATAGGCGAGTACTCTGTTATTGGGACTATTTACGAAGTTATTATAACTTTTTCCTTCTCATACTCATAAGTTGTAAAGGCACAGCGGGAATAAGGGAAAAAAC
[>> AmpR promoter                                                 ][AmpR                                    >>]
       3,540               3,560               3,580               3,600               3,620

CGGCATTTTGCCTTCCTGTTTTTGCTCACCCAGAAACGCTGGTGAAAGTAAAAGATGCTGAAGATCAGTTGGGTGCACGAGTGGGTTACATCGAACTGGATCTCAAC
GCCGTAAAACGGAAGGACAAAAACGAGTGGGTCTTTGCGACCACTTTCATTTTCTACGACTTCTAGTCAACCCACGTGCTCACCCAATGTAGCTTGACCTAGAGTTG
[>>                                          AmpR                                                         >>]
3,640               3,660               3,680               3,700               3,720               3,740

                                      AclI
AGCGGTAAGATCCTTGAGAGTTTTCGCCCCGAAGAACGTTTTCCAATGATGAGCACTTTTAAAGTTCTGCTATGTGGCGCGGTATTATCCCGTATTGACGCCGGGCA
TCGCCATTCTAGGAACTCTCAAAAGCGGGGCTTCTTGCAAAAGGTTACTACTCGTGAAAATTTCAAGACGATACACCGCGCCATAATAGGGCATAACTGCGGCCCGT
[>>                                          AmpR                                                         >>]
             3,760               3,780               3,800               3,820               3,840

                            BcgI                    ScaI
AGAGCAACTCGGTCGCCGCATACACTATTCTCAGAATGACTTGGTTGAGTACTCACCAGTCACAGAAAAGCATCTTACGGATGGCATGACAGTAAGAGAATTATGCA
TCTCGTTGAGCCAGCGGCGTATGTGATAAGAGTCTTACTGAACCAACTCATGAGTGGTCAGTGTCTTTTCGTAGAATGCCTACCGTACTGTCATTCTCTTAATACGT
[>>                                          AmpR                                                         >>]
       3,860               3,880               3,900               3,920               3,940
```

PvuI

GTGCTGCCATAACCATGAGTGATAACACTGCGGCCAACTTACTTCTGACAACGATCGGAGGACCGAAGGAGCTAACCGCTTTTTTGCACAACATGGGGGATCATGTA
CACGACGGTATTGGTACTCACTATTGTGACGCCGGTTGAATGAAGACTGTTGCTAGCCTCCTGGCTTCCTCGATTGGCGAAAAAACGTGTTGTACCCCCTAGTACAT

AmpR

3,960 3,980 4,000 4,020 4,040 4,060

AclI
BsrDI FspI

ACTCGCCTTGATCGTTGGGAACCGGAGCTGAATGAAGCCATACCAAACGACGAGCGTGACACCACGATGCCTGTAGCAATGGCAACAACGTTGCGCAAACTATTAAC
TGAGCGGAACTAGCAACCCTTGGCCTCGACTTACTTCGGTATGGTTTGCTGCTCGCACTGTGGTGCTACGGACATCGTTACCGTTGTTGCAACGCGTTTGATAATTG

AmpR

4,080 4,100 4,120 4,140 4,160

NmeAIII

TGGCGAACTACTTACTCTAGCTTCCCGGCAACAATTAATAGACTGGATGGAGGCGGATAAAGTTGCAGGACCACTTCTGCGCTCGGCCCTTCCGGCTGGCTGGTTTA
ACCGCTTGATGAATGAGATCGAAGGGCCGTTGTTAATTATCTGACCTACCTCCGCCTATTTCAACGTCCTGGTGAAGACGCGAGCCGGGAAGGCCGACCGACCAAAT

AmpR

4,180 4,200 4,220 4,240 4,260 4,280

BsaI BsrDI BmrI AhdI

TTGCTGATAAATCTGGAGCCGGTGAGCGTGGGTCTCGCGGTATCATTGCAGCACTGGGGCCAGATGGTAAGCCCTCCCGTATCGTAGTTATCTACACGACGGGGAGT
AACGACTATTTAGACCTCGGCCACTCGCACCCAGAGCGCCATAGTAACGTCGTGACCCCGGTCTACCATTCGGGAGGGCATAGCATCAATAGATGTGCTGCCCCTCA

AmpR

4,300 4,320 4,340 4,360 4,380

CAGGCAACTATGGATGAACGAAATAGACAGATCGCTGAGATAGGTGCCTCACTGATTAAGCATTGGTAACTGTCAGACCAAGTTTACTCATATATACTTTAGATTGA
GTCCGTTGATACCTACTTGCTTTATCTGTCTAGCGACTCTATCCACGGAGTGACTAATTCGTAACCATTGACAGTCTGGTTCAAATGAGTATATATGAAATCTAACT

AmpR

4,400 4,420 4,440 4,460 4,480

TTTAAAACTTCATTTTTAATTTAAAAGGATCTAGGTGAAGATCCTTTTTGATAATCTCATGACCAAAATCCCTTAACGTGAGTTTTCGTTCCACTGAGCGTCAGACC
AAATTTTGAAGTAAAAATTAAATTTTCCTAGATCCACTTCTAGGAAAAACTATTAGAGTACTGGTTTTAGGGAATTGCACTCAAAAGCAAGGTGACTCGCAGTCTGG

ColE1 origin

4,500 4,520 4,540 4,560 4,580 4,600

CCGTAGAAAAGATCAAAGGATCTTCTTGAGATCCTTTTTTTCTGCGCGTAATCTGCTGCTTGCAAACAAAAAAACCACCGCTACCAGCGGTGGTTTGTTTGCCGGAT
GGCATCTTTTCTAGTTTCCTAGAAGAACTCTAGGAAAAAAAGACGCGCATTAGACGACGAACGTTTGTTTTTTTGGTGGCGATGGTCGCCACCAAACAAACGGCCTA

ColE1 origin

4,620 4,640 4,660 4,680 4,700

CAAGAGCTACCAACTCTTTTTCCGAAGGTAACTGGCTTCAGCAGAGCGCAGATACCAAATACTGTCCTTCTAGTGTAGCCGTAGTTAGGCCACCACTTCAAGAACTC
GTTCTCGATGGTTGAGAAAAAGGCTTCCATTGACCGAAGTCGTCTCGCGTCTATGGTTTATGACAGGAAGATCACATCGGCATCAATCCGGTGGTGAAGTTCTTGAG
ColE1 origin
4,720 4,740 4,760 4,780 4,800

TGTAGCACCGCCTACATACCTCGCTCTGCTAATCCTGTTACCAGTGGCTGCTGCCAGTGGCGATAAGTCGTGTCTTACCGGGTTGGACTCAAGACGATAGTTACCGG
ACATCGTGGCGGATGTATGGAGCGAGACGATTAGGACAATGGTCACCGACGACGGTCACCGCTATTCAGCACAGAATGGCCCAACCTGAGTTCTGCTATCAATGGCC
ColE1 origin
4,820 4,840 4,860 4,880 4,900 4,920

ATAAGGCGCAGCGGTCGGGCTGAACGGGGGGTTCGTGCACACAGCCCAGCTTGGAGCGAACGACCTACACCGAACTGAGATACCTACAGCGTGAGCATTGAGAAAGC
TATTCCGCGTCGCCAGCCCGACTTGCCCCCCAAGCACGTGTGTCGGGTCGAACCTCGCTTGCTGGATGTGGCTTGACTCTATGGATGTCGCACTCGTAACTCTTTCG
ColE1 origin
4,940 4,960 4,980 5,000 5,020

BciVI
GCCACGCTTCCCGAAGGGAGAAAGGCGGACAGGTATCCGGTAAGCGGCAGGGTCGGAACAGGAGAGCGCACGAGGGAGCTTCCAGGGGGAAACGCCTGGTATCTTTA
CGGTGCGAAGGGCTTCCCTCTTTCCGCCTGTCCATAGGCCATTCGCCGTCCCAGCCTTGTCCTCTCGCGTGCTCCCTCGAAGGTCCCCCTTTGCGGACCATAGAAAT
ColE1 origin
5,040 5,060 5,080 5,100 5,120

TAGTCCTGTCGGGTTTCGCCACCTCTGACTTGAGCGTCGATTTTTGTGATGCTCGTCAGGGGGGCGGAGCCTATGGAAAAACGCCAGCAACGCGGCCTTTTTACGGT
ATCAGGACAGCCCAAAGCGGTGGAGACTGAACTCGCAGCTAAAAACACTACGAGCAGTCCCCCCGCCTCGGATACCTTTTTGCGGTCGTTGCGCCGGAAAAATGCCA
ColE1 origin
5,140 5,160 5,180 5,200 5,220 5,240

AflIII
PciI
TCCTGGCCTTTTGCTGGCCTTTTGCTCACATGTTCTTTCCTGCGTTATCCCCTGATTCTGTGGATAACCGTATTACCGCCTTTGAGTGAGCTGATACCGCTCGCCGC
AGGACCGGAAAACGACCGGAAAACGAGTGTACAAGAAAGGACGCAATAGGGGACTAAGACACCTATTGGCATAATGGCGGAAACTCACTCGACTATGGCGAGCGGCG
5,260 5,280 5,300 5,320 5,340

BspQI
SapI BsaXI PvuII
AGCCGAACGACCGAGCGCAGCGAGTCAGTGAGCGAGGAAGCGGAAGAGCGCCCAATACGCAAACCGCCTCTCCCCGCGCGTTGGCCGATTCATTAATGCAGCTGGCA
TCGGCTTGCTGGCTCGCGTCGCTCAGTCACTCGCTCCTTCGCCTTCTCGCGGGTTATGCGTTTGGCGGAGAGGGGCGCGCAACCGGCTAAGTAATTACGTCGACCGT
5,360 5,380 5,400 5,420 5,440

CGACAGGTTTCCCGACTGGAAAGCGGGCAGTGAGCGCAACGCAATTAATGTGAGTTAGCTCACTCATTAGGCACCCCAGGCTTTACACTTTATGCTTCCGGCTCGTA
GCTGTCCAAAGGGCTGACCTTTCGCCCGTCACTCGCGTTGCGTTAATTACACTCAATCGAGTGAGTAATCCGTGGGGTCCGAAATGTGAAATACGAAGGCCGAGCAT
CAP binding site lac promoter
5,460 5,480 5,500 5,520 5,540 5,560

TGTTGTGTGGAATTGTGAGCGGATAACAATTTCACACAGGAAACAGCT
ACAACACACCTTAACACTCGCCTATTGTTAAAGTGTGTCCTTTGTCGA

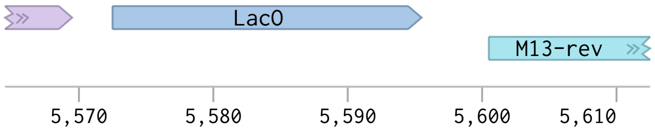

pSTAR (14368 bp)

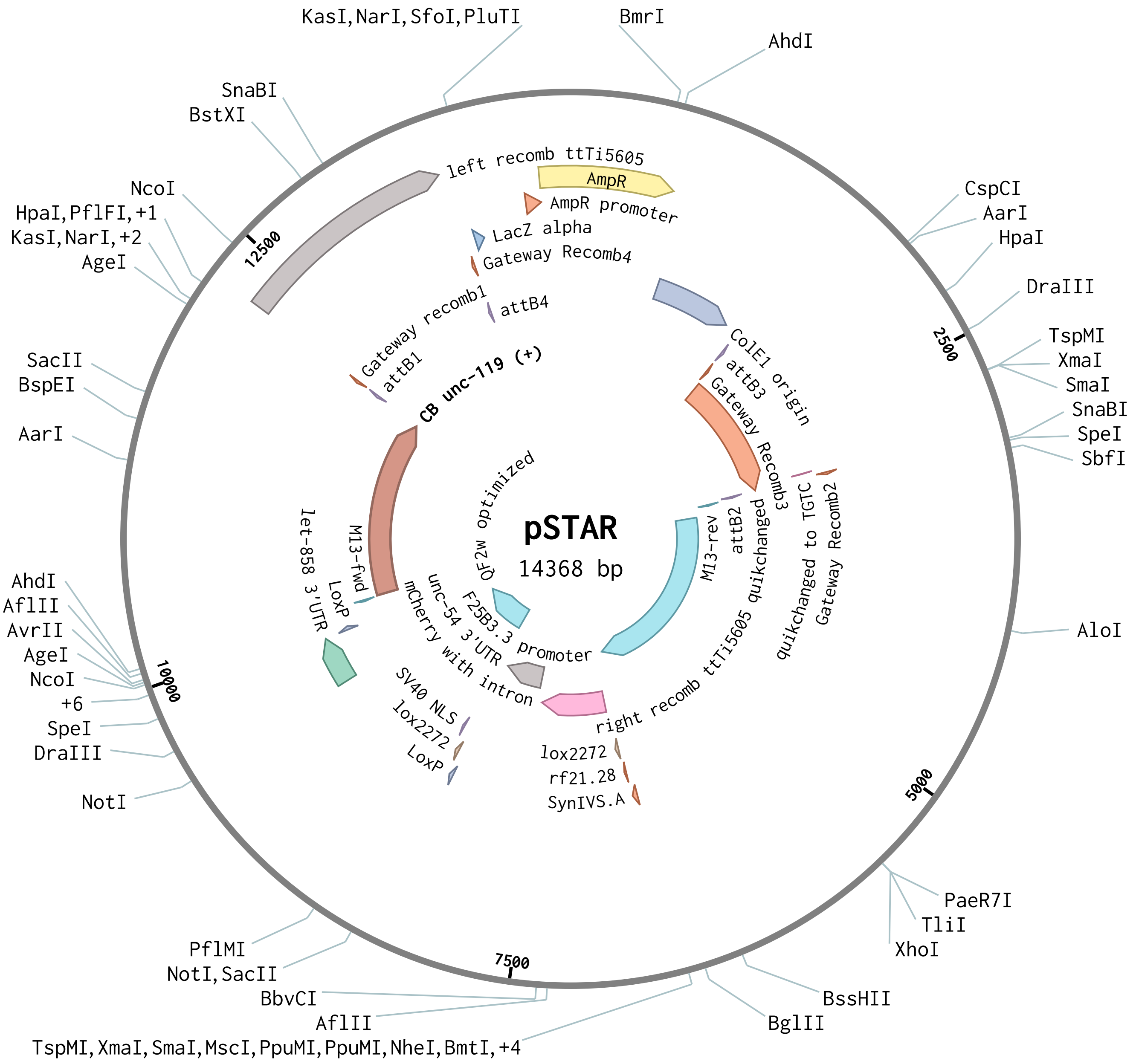

pSTAR (14368 bp)

```
atgagcacttttaaagttctgctatgtggcgcggtattatcccgtattgacgccgggcaagagcaactcggtcgccgcatacactattctcagaatgacttggttga
tactcgtgaaaatttcaagacgatacaccgcgccataatagggcataactgcggcccgttctcgttgagccagcggcgtatgtgataagagtcttactgaaccaact
AmpR
20 40 60 80 100

gtactcaccagtcacagaaaagcatcttacggatggcatgacagtaagagaattatgcagtgctgccataaccatgagtgataacactgcggccaacttacttctga
catgagtggtcagtgtcttttcgtagaatgcctaccgtactgtcattctcttaatacgtcacgacggtattggtactcactattgtgacgccggttgaatgaagact
AmpR
120 140 160 180 200

caacgatcggaggaccgaaggagctaaccgcttttttgcacaacatgggggatcatgtaactcgccttgatcgttgggaaccggagctgaatgaagccataccaaac
gttgctagcctcctggcttcctcgattggcgaaaaaacgtgttgtaccccctagtacattgagcggaactagcaacccttggcctcgacttacttcggtatggtttg
AmpR
220 240 260 280 300 320

gacgagcgtgacaccacgatgcctgtagcaatggcaacaacgttgcgcaaactattaactggcgaactacttactctagcttcccggcaacaattaatagactggat
ctgctcgcactgtggtgctacggacatcgttaccgttgttgcaacgcgtttgataattgaccgcttgatgaatgagatcgaagggccgttgttaattatctgaccta
AmpR
340 360 380 400 420

ggaggcggataaagttgcaggaccacttctgcgctcggcccttccggctggctggtttattgctgataaatctggagccggtgagcgtgggtctcgcggtatcattg
cctccgcctatttcaacgtcctggtgaagacgcgagccgggaaggccgaccgaccaaataacgactatttagacctcggccactcgcacccagagcgccatagtaac
AmpR
440 460 480 500 520

BmrI AhdI
cagcactggggccagatggtaagccctcccgtatcgtagttatctacacgacggggagtcaggcaactatggatgaacgaaatagacagatcgctgagataggtgcc
gtcgtgaccccggtctaccattcgggagggcatagcatcaatagatgtgctgcccctcagtccgttgatacctacttgctttatctgtctagcgactctatccacgg
AmpR
540 560 580 600 620 640

tcactgattaagcattggtaactgtcagaccaagtttactcatatatactttagattgatttaaaacttcatttttaatttaaaaggatctaggtgaagatcctttt
agtgactaattcgtaaccattgacagtctggttcaaatgagtatatatgaaatctaactaaattttgaagtaaaaattaaattttcctagatccacttctaggaaaa
AmpR
660 680 700 720 740

tgataatctcatgaccaaaatcccttaacgtgagttttcgttccactgagcgtcagaccccgtagaaaagatcaaaggatcttcttgagatcctttttttctgcgcg
actattagagtactggttttagggaattgcactcaaaagcaaggtgactcgcagtctggggcatcttttctagtttcctagaagaactctaggaaaaaaagacgcgc
ColE1 origin
760 780 800 820 840
```

taatctgctgcttgcaaacaaaaaaaccaccgctaccagcggtggtttgtttgccggatcaagagctaccaactctttttccgaaggtaactggcttcagcagagcg
attagacgacgaacgtttgtttttttggtggcgatggtcgccaccaaacaaacggcctagttctcgatggttgagaaaaaggcttccattgaccgaagtcgtctcgc

ColE1 origin

860 880 900 920 940 960

cagataccaaatactgtccttctagtgtagccgtagttaggccaccacttcaagaactctgtagcaccgcctacatacctcgctctgctaatcctgttaccagtggc
gtctatggtttatgacaggaagatcacatcggcatcaatccggtggtgaagttcttgagacatcgtggcggatgtatggagcgagacgattaggacaatggtcaccg

ColE1 origin

980 1,000 1,020 1,040 1,060

tgctgccagtggcgataagtcgtgtcttaccgggttggactcaagacgatagttaccggataaggcgcagcggtcgggctgaacggggggttcgtgcacacagccca
acgacggtcaccgctattcagcacagaatggcccaacctgagttctgctatcaatggcctattccgcgtcgccagcccgacttgccccccaagcacgtgtgtcgggt

ColE1 origin

1,080 1,100 1,120 1,140 1,160

gcttggagcgaacgacctacaccgaactgagatacctacagcgtgagctatgagaaagcgccacgcttcccgaagggagaaaggcggacaggtatccggtaagcggc
cgaacctcgcttgctggatgtggcttgactctatggatgtcgcactcgatactctttcgcggtgcgaagggcttccctctttccgcctgtccataggccattcgccg

ColE1 origin

1,180 1,200 1,220 1,240 1,260 1,280

agggtcggaacaggagagcgcacgagggagcttccagggggaaacgcctggtatctttatagtcctgtcgggtttcgccacctctgacttgagcgtcgatttttgtg
tcccagccttgtcctctcgcgtgctccctcgaaggtccccctttgcggaccatagaaatatcaggacagcccaaagcggtggagactgaactcgcagctaaaaacac

ColE1 origin

1,300 1,320 1,340 1,360 1,380

atgctcgtcaggggggcggagcctatggaaaaacgccagcaacgcggcctttttacggttcctggtcttttgctggccttttgctcacatgttctttcctgcgttat
tacgagcagtccccccgcctcggatacctttttgcggtcgttgcgccggaaaaatgccaaggaccagaaaacgaccggaaaacgagtgtacaagaaaggacgcaata

ColE1 origin

1,400 1,420 1,440 1,460 1,480

cccctgattctgtggataaccgtattaccgcctttgagtgagctgataccATCgaattatcaactatgtataataaagttgtgtttttgaacacggcgatatgtatc
ggggactaagacacctattggcataatggcggaaactcactcgactatggTAGcttaatagttgatacatattatttcaacacaaaaacttgtgccgctatacatag

Gateway Recomb3

attB3

1,500 1,520 1,540 1,560 1,580 1,600

tgtagatcatccccaattttcaacaatgaatctaaatactgaactttttgtgtaatctttagtgcagaaaaatgaccaaaatcagcgattttccgattatacccaaa
acatctagtaggggttaaaagttgttacttagatttatgacttgaaaaacacattagaaatcacgtctttttactggttttagtcgctaaaaggctaatatgggttt

right recomb ttTi5605 quikchanged

1,620 1,640 1,660 1,680 1,700

atatctcagctcaatgagcacctataaaaatgtaaaaataaagaaagtagtcgaaccatatttgtaaaggctaaaaaactgaataaatttttatccatccagcgcg
tatagagtcgagttactcgtggatatttttacatttttatttctttcatcagcttggtataaacatttccgatttttgacttatttaaaaataggtaggtcgcgc
right recomb ttTi5605 quikchanged
1,720 1,740 1,760 1,780 1,800

gcgttacaatccgtggcccatgtcacgcacggctttcaaagaaaaaaaaacagtctgtttgcgcgacatcgatctggcaaaaatcctaaaaacgactatgcgtggct
cgcaatgttaggcaccgggtacagtgcgtgccgaaagtttcttttttttgtcagacaaacgcgctgtagctagaccgtttttaggatttttgctgatacgcaccga
right recomb ttTi5605 quikchanged
1,820 1,840 1,860 1,880 1,900 1,920

CspCI AarI
cactttatattaatatgaaaaatacaaacttggtggtgcaggtgctgcagaggccatcgatgaagaagtggaggccttgttgtcgatctacaaaattgtatggttgt
gtgaaatataattatactttttatgtttgaaccaccacgtccacgacgtctccggtagctacttcttcacctccggaacaacagctagatgttttaacataccaaca
right recomb ttTi5605 quikchanged
1,940 1,960 1,980 2,000 2,020

ttgagattttccttgaaacgctctcaatcttggatatgatataaattctggataagatatgatggatatgatctatgtttgatagagtgcatttgaaaggataacgg
aactctaaaaggaactttgcgagagttagaacctatactatatttaagacctattctatactacctatactagatacaaactatctcacgtaaactttcctattgcc
right recomb ttTi5605 quikchanged
2,040 2,060 2,080 2,100 2,120 2,140

ccctgcagacatttttgaaaaaataacaaacatttaattttctcactcgtttaggctattcccctatttttgatattccttcgcacatatgaaaactactttttttc
gggacgtctgtaaaaactttttttattgtttgtaaattaaaagagtgagcaaatccgataaggggataaaaactataaggaagcgtgtatactttgatgaaaaaaag
right recomb ttTi5605 quikchanged
2,160 2,180 2,200 2,220 2,240

HpaI
gaaactgttaactccagaatttataaatctatagcccttacttgattatttattatcatggttactcacctcatgtctctttctcttttcaagaagtcttcgttcat
ctttgacaattgaggtcttaaatatttagatatcgggaatgaactaataaataatagtaccaatgagtggagtacagagaaagagaaaagttcttcagaagcaagta
right recomb ttTi5605 quikchanged
2,260 2,280 2,300 2,320 2,340

atctgttgtctctttcctccttttgaatctctgctttgctcttcgccattgtttcctgaaaataatgtaacttgaattgtgtaatatactttttttaatttgaatttg
tagacaacagagaaaggaggaaaacttagagacgaaacgagaagcggtaacaaaggacttttattacattgaacttaacacattatatgaaaaaattaaacttaaac
right recomb ttTi5605 quikchanged
2,360 2,380 2,400 2,420 2,440 2,460

DraIII
gcttgtaacgcggaatcactacgtgcgggatcatttcttactagaaaacccagaaaatgccatatttcacttatctcggggtcatttctaattagaaaagctacaaa
cgaacattgcgccttagtgatgcacgccctagtaaagaatgatcttttgggtcttttacggtataaagtgaatagagccccagtaaagattaatcttttcgatgttt
right recomb ttTi5605 quikchanged
2,480 2,500 2,520 2,540 2,560

```
aaccatttctaattacggtgactttagaactggtcgaaatagctaattagaaatgggtttttgctaattagaggtgactctatgttatctgagtaagcctcgtttgt
ttggtaaagattaatgccactgaaatcttgaccagctttatcgattaatctttacccaaaaacgattaatctccactgagatacaatagactcattcggagcaaaca
```

right recomb ttTi5605 quikchanged

2,580 2,600 2,620 2,640 2,660

SmaI
TspMI
XmaI

```
atgtaaactcacagtactaataaatgcaagacacccgggtttgtctagatatgaaataattgaaatatcaattctgacagacaataatggtaatcttgataaggagt
tacatttgagtgtcatgattatttacgttctgtgggcccaaacagatctatactttattaactttatagttaagactgtctgttattaccattagaactattcctca
```

right recomb ttTi5605 quikchanged

2,680 2,700 2,720 2,740 2,760 2,780

```
tccacgcccaggagaacacgttagttttcttgtttttgattgcgtgcgttattttggagaaaaactcgatttttttacaaaataatttttttgaaaggaacactgttca
aggtgcgggtcctcttgtgcaatcaaaagaacaaaaactaacgcacgcaataaaacctctttttgagctaaaaaatgttttattaaaaactttccttgtgacaagt
```

right recomb ttTi5605 quikchanged

2,800 2,820 2,840 2,860 2,880

```
ataagttttgtctttttttctcagttgtgatacggtttttattctttttttgtagttatacagaagaccgttacgaaTGTCactgatatcgaaacaaacactgatctt
tattcaaaacagaaaaaagagtcaacactatgccaaaaaataagaaaaaacatcaatatgtcttctggcaatgcttACAGtgactatagctttgtttgtgactagaa
```

right recomb ttTi5605 quikchanged

2,900 2,920 2,940 2,960 2,980

SnaBI SpeI

```
acttgcacttaccactttgtacaagAAAGCTGGGTcacgggCAGGAAACAGCTATGACCATGATTACGCCAAGCTACGTAATACGACTCACTAGTGGGCAGATCACC
tgaacgtgaatggtgaaacatgttcTTTCGACCCAgtgcccGTCCTTTGTCGATACTGGTACTAATGCGGTTCGATGCATTATGCTGAGTGATCACCCGTCTAGTGG
```

attB2
Gateway Recomb2 M13-rev

3,000 3,020 3,040 3,060 3,080 3,100

SbfI

```
ATGATTACGCCGCATGCCTGCAGGTCGACTCTAGAGGATCAAGAGCATTTGAATCAGAATATGGAGAACGGAGCATGAGCATTTTCGAAGTTTTTTAGATGGATCCg
TACTAATGCGGCGTACGGACGTCCAGCTGAGATCTCCTAGTTCTCGTAAACTTAGTCTTATACCTCTTGCCTCGTACTCGTAAAAGCTTCAAAAAATCTACCTAGGc
```

3,120 3,140 3,160 3,180 3,200

```
catgcaagactaattttcgattaacccgtaggggtgcaagactaatagagactgcaagactattagaggctgaaatactaattttcgtatgctcaataattttggaa
gtacgttctgattaaaagctaattgggcatccccacgttctgattatctctgacgttctgataatctccgactttatgattaaaagcatacgagttattaaaacctt
```

F25B3.3 promoter

3,220 3,240 3,260 3,280 3,300

attggcctatttttttgtagaaacttgataccgtttaaacaaggaaaaatacacactttttaatatttcatcaataatttgaacgattttgtgattttaagttcaatt
taaccggataaaaaacatctttgaactatggcaaatttgttcctttttatgtgtgaaaaattataaagtagttattaaacttgctaaaacactaaaattcaagttaa
F25B3.3 promoter
3,320 3,340 3,360 3,380 3,400 3,420

tgcccaaaaaaagacaatttttctgacgttaccgcaaagtaatgctggtcaggcaaaaagagcggtgcaaaaatataagagactgcaatactaatagaggaaatacg
acgggtttttttctgttaaaaagactgcaatggcgtttcattacgaccagtccgtttttctcgccacgtttttatattctctgacgttatgattatctcctttatgc
F25B3.3 promoter
3,440 3,460 3,480 3,500 3,520

gtaattgaattttagtgaaacttgcgacagttttcctcatttttttgttattccggcatacgagtgtggcatacgagtgaggtcatcttttgtttcttccgtttctt
cattaacttaaaatcactttgaacgctgtcaaaaggagtaaaaaaacaataaggccgtatgctcacaccgtatgctcactccagtagaaaacaaagaaggcaaagaa
F25B3.3 promoter
3,540 3,560 3,580 3,600 3,620

catcgcttttgaaaaaaaatgttgaagaaaacttcgtagccgatagccggataatgtttggcacggcgttccaacatatatcattggaaatttttttaagatttttgc
gtagcgaaaacttttttttacaacttcttttgaagcatcggctatcggcctattacaaaccgtgccgcaaggttgtatatagtaacctttaaaaaattctaaaaacg
F25B3.3 promoter
3,640 3,660 3,680 3,700 3,720 3,740

gaaaaatcacattcttcacgatgagaacacgttattgaaggaatataaatcaagaataacatatagttatatttctctattactttaacgttaaatatgagcaaatt
ctttttagtgtaagaagtgctactcttgtgcaataacttccttatatttagttcttattgtatatcaatataaagagataatgaaattgcaatttatactcgtttaa
F25B3.3 promoter
3,760 3,780 3,800 3,820 3,840

tgagcattttgattgcgatgaaaagcagaatcgagtcaactgaaatccgttcaaaaacctaagctcggttcgcccgaaagtcgatttttccaatagccgaacagcac
actcgtaaaactaacgctacttttcgtcttagctcagttgactttaggcaagtttttggattcgagccaagcgggctttcagctaaaaaggttatcggcttgtcgtg
F25B3.3 promoter
3,860 3,880 3,900 3,920 3,940

AloI
tgcttctttattttccaggtatttttggggtgtaagaattatatcttctttgtttgggtatttacgtttccgaattccctttcttgatctgtcgctatttttcagaa
acgaagaaataaaaggtccataaaaaccccacattcttaatatagaagaaacaaacccataaatgcaaaggcttaagggaaagaactagacagcgataaaaagtctt
F25B3.3 promoter
3,960 3,980 4,000 4,020 4,040 4,060

accagaacagtttatcctttttcatgagaacctgaatagctcaaaaacccgtttaatttcttttcatcatctcatttgaaactttcctagtcttcttaatgatttcc
tggtcttgtcaaataggaaaaagtactcttggacttatcgagtttttgggcaaattaaagaaaagtagtagagtaaactttgaaaggatcagaagaattactaaagg
F25B3.3 promoter
4,080 4,100 4,120 4,140 4,160

aggctcctcctacatttgtatctcaagatgcatacactttatttccgctccaattcgaatcatctacttctctttttttcttcaactttctactttttttccattcttt
tccgaggaggatgtaaacatagagttctacgtatgtgaaataaaggcgaggttaagcttagtagatgaagagaaaaaaagaagttgaaagatgaaaaaaaggtaagaaa

F25B3.3 promoter

4,180 4,200 4,220 4,240 4,260 4,280

tctttcaaatgttcgacgtcttcaagtgactgctcttcattctcctcatttttctcgaatttcattcttgtctctttttttgctcaaaaatggaaaatgaaagtgacg
agaaagtttacaagctgcagaagttcactgacgagaagtaagaggagtaaaaagagcttaaagtaagaacagagaaaaaaacgagtttttaccttttactttcactgc

F25B3.3 promoter

4,300 4,320 4,340 4,360 4,380

tgagatttggacggcgggacacgggggcagtagaagcagcaaaaaggagagaaagaggacacaaataagaagaacgaattcaaaaataagcggagaggagctatttc
actctaaacctgccgccctgtgcccccgtcatcttcgtcgtttttcctctctttctcctgtgtttattcttcttgcttaagtttttattcgcctctcctcgataaag

F25B3.3 promoter

4,400 4,420 4,440 4,460 4,480

cgtcaattctacctccccaatcttcatcaattcggctcaattgaatgacgtcacagggagataaaccgtttggatgagcgccgtccatcactgacgccatcccgttt
gcagttaagatggaggggttagaagtagttaagccgagttaacttactgcagtgtccctctatttggcaaacctactcgcggcaggtagtgactgcggtagggcaaa

F25B3.3 promoter

4,500 4,520 4,540 4,560 4,580 4,600

gggacaagaaaagagaaaaaagagcacaaagtttttggtgacggatcttgtcaatcatatgaaagttgttctgattgattgtcagtttttttcctactttttttggat
ccctgttcttttctcttttttctcgtgtttcaaaaaccactgcctagaacagttagtatactttcaacaagactaactaacagtcaaaaaaaggatgaaaaaaaccta

F25B3.3 promoter

4,620 4,640 4,660 4,680 4,700

tctatccacttctgaacttttgacaagtttcaaactttctgaatcattttctatgcattttcctggaattctttttatgtaaaatatgaaatagaatgtttttgaat
agataggtgaagacttgaaaactgttcaaagtttgaaagacttagtaaaagatacgtaaaaggaccttaagaaaaatacattttatactttatcttacaaaaactta

F25B3.3 promoter

4,720 4,740 4,760 4,780 4,800

tcaagttctgctttttttcttctttttttgttctgttcgggcttgggtatgcttttttttaaaaattattttgcacatcgaccaataagtgcgcaacttataaaattaat
agttcaagacgaaaaaaagaagaaaaaaacaagacaagcccgaacccatacgaaaaaaaatttttaataaaacgtgtagctggttattcacgcgttgaatattttaatta

F25B3.3 promoter

4,820 4,840 4,860 4,880 4,900 4,920

ttatttttgttaatttttgaaatacttgtattgctttaagtgatctgacctcgccctgagctttccacgtagttatcaaatacaaatcctccaagggtaacgtacct
aataaaaacaattaaaaactttatgaacataacgaaattcactagactggagcgggactcgaaaggtgcatcaatagtttatgtttaggaggttcccattgcatgga

F25B3.3 promoter

4,940 4,960 4,980 5,000 5,020

atattactgatctttataataactttatcacctgtccagttccagaggaattctgttaagcttataggcacagaaggagtcatttctgctggtttttaatgatccaa
tataatgactagaaatattattgaaatagtggacaggtcaaggtctccttaagacaattcgaatatccgtgtcttcctcagtaaagacgaccaaaaattactaggtt
F25B3.3 promoter
5,040 5,060 5,080 5,100 5,120

atctttatttcaagtaaaaaactgaacacttgcgaataaaactatcagattaaccattcaccaaaaatgtgtttgaatctaaaactttctcagtattccaaatatag
tagaaataaagttcattttttgacttgtgaacgcttattttgatagtctaattggtaagtggtttttacacaaacttagattttgaaagagtcataaggtttatatc
F25B3.3 promoter
5,140 5,160 5,180 5,200 5,220 5,240

aaataaataaccacgacattgctaaaatctgtctgaattgtgtactccttaccgtgaagtaataatggatataatgaatcgtttgaaatgaatgatcagcacatttt
tttatttattggtgctgtaacgattttagacagacttaacacatgaggaatggcacttcattattacctatattacttagcaaactttacttactagtcgtgtaaaa
F25B3.3 promoter
5,260 5,280 5,300 5,320 5,340

PaeR7I
XhoI
TliI
tggtgaaagatcacaaataaggaataagcgacggaaaataaaacgattatttcggatcaaaaatttgttgaagatcatatacactcgagaccaagattattctagac
accactttctagtgtttattccttattcgctgccttttattttgctaataaagcctagtttttaaacaacttctagtatatgtgagctctggttctaataagatctg
F25B3.3 promoter
5,360 5,380 5,400 5,420 5,440

aattttcaaattggcttctttgtttgcaaatcttcataataatctgtgaagtttggaaatttgaattttaatctttttcatagattatagtttttatttctttgc
ttaaaagtttaaccgaagaaacaaacgtttagaagtattattagacacttcaaacctttaaacttaaaattagaaaaagtatctaatatcaaaaataaagaaacg
F25B3.3 promoter
5,460 5,480 5,500 5,520 5,540 5,560

aaaactatattaaaaaccgatgcattgttttagggaaattaatgagccttttgttcaacactaaaaacaataaaattaaaattttggcttcatcatttgaccttttt
ttttgatataatttttggctacgtaacaaaatccctttaattactcggaaaacaagttgtgatttttgttattttaattttaaaaccgaagtagtaaactggaaaaa
F25B3.3 promoter
5,580 5,600 5,620 5,640 5,660

taagttcgaaaacttttctcgtatttctgaaccgccaatttttcacacatctctagacttttggtgcccgttccagaaagttaagtaattgctattctaagaaagt
attcaagcttttgaaaagagcataaagacttggcggttaaaaagtgtgtagagatctgaaaaccacgggcaaggtctttcaattcattaacgataagattctttca
F25B3.3 promoter
5,680 5,700 5,720 5,740 5,760

tctcaacattgtttttttagttctgattgaattctgatgttccaggaatatattttaaatttaatattctttgcactatttctatactaactaaataataaatagtct
agagttgtaacaaaaaaatcaagactaacttaagactacaaggtccttatataaaatttaaattataagaaacgtgataaagatatgattgatttattatttatcaga
F25B3.3 promoter
5,780 5,800 5,820 5,840 5,860 5,880

```
aagtatgttcaatgagaacaaaaaagctctatctatatttttctatcctatttcatttatatccttttcattttgaactcaccagcttctcttcttcttcttgtcca
ttcatacaagttactcttgttttttcgagatagatataaaaagataggataaagtaaatataggaaaagtaaaacttgagtggtcgaagagaagaagaagaacaggt
                                    F25B3.3 promoter
        5,900               5,920               5,940               5,960               5,980

tatacttcttaaaagtctcgaacttctctctctcactctccatcaatttctttatggctaccgcttgcgctctgccgcttccgaagaaagaaggcgttggcagagct
atatgaagaattttcagagcttgaagagagagagtgagaggtagttaaagaaataccgatggcgaacgcgagacggcgaaggcttctttcttccgcaaccgtctcga
                                    F25B3.3 promoter
     6,000               6,020               6,040               6,060               6,080

ctcaattctatttttttctcgccgtaggctatcgatttctcagcttctcttctctcttcctcactccaccaccaccacttcgggcttcttcttttcttcattttcgtc
gagttaagataaaaaagagcggcatccgatagctaaagagtcgaagagaagagagaaggagtgaggtggtggtggtgaagcccgaagaagaaaagaagtaaaagcag
                                    F25B3.3 promoter
6,100               6,120               6,140               6,160               6,180               6,200

                                                                                     BssHII
ttcttcttcatcctcttctttttttttcagttccccctcactcctccccttatatgcgcgtgcgagaggggtgcaaaagcagcgcgcatccgagaattgaaacgaga
aagaagaagtaggagaagaaaaaaaaagtcaagggggagtgaggaggggaatatacgcgcacgctctccccacgttttcgtcgcgcgtaggctcttaactttgctct
                                    F25B3.3 promoter
         6,220               6,240               6,260               6,280               6,300

aaacggagacgcagcagcagttcgtcctcagaaaaatagccagtaaaaagagaaaaagatagagagagacctatcgcattttattttcaaattgcaattcctgcaat
tttgcctctgcgtcgtcgtcaagcaggagtctttttatcggtcatttttctctttttctatctctctctggatagcgtaaaataaaagtttaacgttaaggacgtta
                                    F25B3.3 promoter
    6,320               6,340               6,360               6,380               6,400              6,420

                                                                        BglII
tcatgtgtgcgtgtgcccattttcaattcttcccgttatttttcagaccattaccaacgttctttctagatcttgagacaatcttccttctgctcaatcgttcgtcg
agtacacacgcacacgggtaaaagttaagaagggcaataaaaagtctggtaatggttgcaagaaagatctagaactctgttagaaggaagacgagttagcaagcagc
                                    F25B3.3 promoter
             6,440               6,460               6,480               6,500               6,520

                                                       SmaI
                                                     TspMI
                                                     XmaI        MscI  PpuMI
tgaagacggcatcgacgacgacgacgatCagcagtaaagttgaGGATCCCCGGGATTGGCCAAAGGACCCAAAGGTATGTTTCGAATGATACTAACATAACATAGAA
acttctgccgtagctgctgctgctgctaGtcgtcatttcaactCCTAGGGGCCCTAACCGGTTTCCTGGGTTTCCATACAAAGCTTACTATGATTGTATTGTATCTT
            F25B3.3 promoter                                               SynIVS.A
        6,540               6,560               6,580               6,600               6,620
```

PpuMI NheI BmtI SgrDI BglII

CATTTTCAGGAGGACCCTTGGCTAGCGTCGACGGTACCATAACTTCGTATAGGATACTTTATACGAAGTTATAACGTTAGATCTATGGTCTCAAAGGGTGAAGAAGA
GTAAAAGTCCTCCTGGGAACCGATCGCAGCTGCCATGGTATTGAAGCATATCCTATGAAATATGCTTCAATATTGCAATCTAGATACCAGAGTTTCCCACTTCTTCT

rf21.28 lox2272

6,640 6,660 6,680 6,700 6,720 6,740

TAACATGGCAATTATTAAAGAGTTTATGCGTTTCAAGGTGCATATGGAGGGATCTGTCAATGGGCATGAGTTTGAAATTGAAGGTGAAGGAGAAGGCCGACCATATG
ATTGTACCGTTAATAATTTCTCAAATACGCAAAGTTCCACGTATACCTCCCTAGACAGTTACCCGTACTCAAACTTTAACTTCCACTTCCTCTTCCGGCTGGTATAC

mCherry with intron

6,760 6,780 6,800 6,820 6,840

BstXI

AGGGAACACAAACCGCAAAACTAAAGgtaagtttaaacatatatatactaactaaccctgattatttaaattttcagGTAACTAAAGGCGGACCATTACCATTCGCC
TCCCTTGTGTTTGGCGTTTTGATTTCcattcaaatttgtatatatatgattgattgggactaataaatttaaaagtcCATTGATTTCCGCCTGGTAATGGTAAGCGG

mCherry with intron

6,860 6,880 6,900 6,920 6,940

TGGGACATCCTCTCTCCACAGTTCATGTATGGAAGTAAAGCTTATGTTAAACATCCGGCAGATATACCAGATTATTTGAAACTTTCATTCCCGGAGGGTTTTAAGTG
ACCCTGTAGGAGAGAGGTGTCAAGTACATACCTTCATTTCGAATACAATTTGTAGGCCGTCTATATGGTCTAATAAACTTTGAAAGTAAGGGCCTCCCAAAATTCAC

mCherry with intron

6,960 6,980 7,000 7,020 7,040 7,060

MluI

GGAACGCGTAATGAATTTTGAAGACGGAGGAGTTGTTACAGTGACGCAAGACTCAAGgtaagtttaaacagttcggtactaactaaccatacatatttaaattttca
CCTTGCGCATTACTTAAAACTTCTGCCTCCTCAACAATGTCACTGCGTTCTGAGTTCcattcaaatttgtcaagccatgattgattggtatgtataaatttaaaagt

mCherry with intron

7,080 7,100 7,120 7,140 7,160

gCCTCCAAGATGGAGAATTTATTTATAAAGTCAAACTTCGAGGAACGAATTTCCCCTCGGATGGACCTGTTATGCAGAAGAAGACTATGGGATGGGAAGCTTCAAGT
cGGAGGTTCTACCTCTTAAATAAATATTTCAGTTTGAAGCTCCTTGCTTAAAGGGGAGCCTACCTGGACAATACGTCTTCTTCTGATACCCTACCCTTCGAAGTTCA

mCherry with intron

7,180 7,200 7,220 7,240 7,260

AflII BbvCI

GAAAGAATGTACCCTGAAGACGGTGCTCTTAAGGGAGAGATTAAACAACGTCTTAAATTGAAAGATGGAGGACATTACGATGCTGAGgtaagtttaaacatgatttt
CTTTCTTACATGGGACTTCTGCCACGAGAATTCCCTCTCTAATTTGTTGCAGAATTTAACTTTCTACCTCCTGTAATGCTACGACTCcattcaaatttgtactaaaa

mCherry with intron

7,280 7,300 7,320 7,340 7,360 7,380

actaactaactaatctgatttaaattttcagGTGAAGACAACTTACAAAGCCAAAAAACCAGTTCAGCTGCCAGGAGCGTACAATGTTAATATTAAACTGGATATCA
tgattgattgattagactaaatttaaaagtcCACTTCTGTTGAATGTTTCGGTTTTTTGGTCAAGTCGACGGTCCTCGCATGTTACAATTATAATTTGACCTATAGT
mCherry with intron
7,400 7,420 7,440 7,460 7,480

CCTCCCACAACGAGGATTACACTATCGTTGAGCAATATGAAAGAGCTGAAGGGCGGCACTCGACAGGTGGCATGGATGAATTGTATAAGTAGGAGCTCCGCATCGGC
GGAGGGTGTTGCTCCTAATGTGATAGCAACTCGTTATACTTTCTCGACTTCCCGCCGTGAGCTGTCCACCGTACCTACTTAACATATTCATCCTCGAGGCGTAGCCG
mCherry with intron
7,500 7,520 7,540 7,560 7,580

CGCTGTCATCAGATCGCCATCTCGCGCCCGTGCCTCTGACTTCTAAGTCCAATTACTCTTCAACATCCCTACATGCTCTTTCTCCCTGTGCTCCCACCCCCTATTTT
GCGACAGTAGTCTAGCGGTAGAGCGCGGGCACGGAGACTGAAGATTCAGGTTAATGAGAAGTTGTAGGGATGTACGAGAAAGAGGGACACGAGGGTGGGGGATAAAA
unc-54 3'UTR
7,600 7,620 7,640 7,660 7,680 7,700

TGTTATTATCAAAAAACTTCTCTTAATTTCTTTGTTTTTTAGCTTCTTTTAAGTCACCTCTAACAATGAAATTGTGTAGATTCAAAAATAGAATTAATTCGTAATAA
ACAATAATAGTTTTTTGAAGAGAATTAAAGAAACAAAAAATCGAAGAAAATTCAGTGGAGATTGTTACTTTAACACATCTAAGTTTTTATCTTAATTAAGCATTATT
unc-54 3'UTR
7,720 7,740 7,760 7,780 7,800

AAAGTCGAAAAAAATTGTGCTCCCTCCCCCCATTAATAATAATTCTATCCCAAAATCTACACAATGTTCTGTGTACACTTCTTATGTTTTTTACTTCTGATAAATTT
TTTCAGCTTTTTTTAACACGAGGGAGGGGGGTAATTATTATTAAGATAGGGTTTTAGATGTGTTACAAGACACATGTGAAGAATACAAAAAATGAAGACTATTTAAA
unc-54 3'UTR
7,820 7,840 7,860 7,880 7,900

TTTTGAAACATCATAGAAAAAACCGCACACAAAATACCTTATCATATGTTACGTTTCAGTTTATGACCGCAATTTTTATTTCTTCGCACGTCTGGGCCTCTCATGAC
AAAACTTTGTAGTATCTTTTTTGGCGTGTGTTTTATGGAATAGTATACAATGCAAAGTCAAATACTGGCGTTAAAAATAAAGAAGCGTGCAGACCCGGAGAGTACTG
unc-54 3'UTR
7,920 7,940 7,960 7,980 8,000 8,020

GTCAAATCATGCTCATCGTGAAAAAGTTTTGGAGTATTTTTGGAATTTTTCAATCAAGTGAAAGTTTATGAAATTAATTTTCCTGCTTTTGCTTTTTGGGGTTTCCC
CAGTTTAGTACGAGTAGCACTTTTTCAAAACCTCATAAAAACCTTAAAAAGTTAGTTCACTTTCAAATACTTTAATTAAAAGGACGAAAACGAAAAACCCCAAAGGG
unc-54 3'UTR
8,040 8,060 8,080 8,100 8,120

CTATTGTTTGTCAAGATTTCGAGGACGGCGTTTTTCTTGCTAAAATCACAAGTATTGATGAGCACGATGCAAGAAAGATCGGAAGAAGGTTTGGGTTTGAGAGGCCT
GATAACAAACAGTTCTAAAGCTCCTGCCGCAAAAAGAACGATTTTAGTGTTCATAACTACTCGTGCTACGTTCTTTCTAGCCTTCTTCCAAACCCAAACTCTCCGGA
unc-54 3'UTR
8,140 8,160 8,180 8,200 8,220

ATAACTTCGTATAGCATACATTATACGAAGTTATACCGGCTAGTGATTCGCCACCATAACTTCGTATAGGATACTTTATACGAAGTTATTGATCAATTCACAGCCAC
TATTGAAGCATATCGTATGTAATATGCTTCAATATGGCCGATCACTAAGCGGTGGTATTGAAGCATATCCTATGAAATATGCTTCAATAACTAGTTAAGTGTCGGTG

LoxP lox2272

8,240 8,260 8,280 8,300 8,320 8,340

SacII
NotI

CGCGGCCGCATGCCACCAAAAAAAAAAAGAAAAGTTGGATCAGGATCAGGATCAGGATCAGGATCAGGAACACCACCAAAAAGAAAAACACTTAATGCTGCTGCTGA
GCGCCGGCGTACGGTGGTTTTTTTTTTTCTTTTCAACCTAGTCCTAGTCCTAGTCCTAGTCCTAGTCCTTGTGGTGGTTTTTCTTTTTGTGAATTACGACGACGACT

SV40 NLS QF2w optimized

8,360 8,380 8,400 8,420 8,440

AGCTAATGCTCATGCTGATGGACATGCTGATGGAAATGCTGATGGACATGTTGCTAATACAGCTGCTTCATCAAATAATGCTAGATTCGCTGATCTTACAAATATTG
TCGATTACGAGTACGACTACCTGTACGACTACCTTTACGACTACCTGTACAACGATTATGTCGACGAAGTAGTTTATTACGATCTAAGCGACTAGAATGTTTATAAC

QF2w optimized

8,460 8,480 8,500 8,520 8,540 8,560

PflMI

ATACACCAGGACTTGGACCAACAACAACAACACTTCTTGTTGAACCAGCTAGATCAAAAAGACAAAGAGTTTCAAGAGCTTGTGATCAATGTAGAGCTGCTAGAGAA
TATGTGGTCCTGAACCTGGTTGTTGTTGTTGTGAAGAACAACTTGGTCGATCTAGTTTTTCTGTTTCTCAAAGTTCTCGAACACTAGTTACATCTCGACGATCTCTT

QF2w optimized

8,580 8,600 8,620 8,640 8,660

AAATGTGATGGAATTCAACCAGCTTGTTTCCCATGTGTTTCACAAGGAAGATCATGTACATATCAAGCTTCACCAAAAAAAAGAGGAGTTCAAACAGGATATATTAG
TTTACACTACCTTAAGTTGGTCGAACAAAGGGTACACAAAGTGTTCCTTCTAGTACATGTATAGTTCGAAGTGGTTTTTTTTCTCCTCAAGTTTGTCCTATATAATC

QF2w optimized

8,680 8,700 8,720 8,740 8,760

AACACTTGAACTTGCTCTTGCTTGGATGTTCGAAAATGTTGCTAGATCAGAAGATGCTCTTCATAATCTTCTTGTTAGAGATGCTGGACAAGGATCAGCTCTTCTTG
TTGTGAACTTGAACGAGAACGAACCTACAAGCTTTTACAACGATCTAGTCTTCTACGAGAAGTATTAGAAGAACAATCTCTACGACCTGTTCCTAGTCGAGAAGAAC

QF2w optimized

8,780 8,800 8,820 8,840 8,860 8,880

TTGGAAAAGATTCACCAGCTGCTGAAAGACTTCATGCTAGATGGGCTACATCAAGAGTTAATAAATCAATTACAAGACTTCTTAGACAACTTGAACTTCCACCAACA
AACCTTTTCTAAGTGGTCGACGACTTTCTGAAGTACGATCTACCCGATGTAGTTCTCAATTATTTAGTTAATGTTCTGAAGAATCTGTTGAACTTGAAGGTGGTTGT

QF2w optimized

8,900 8,920 8,940 8,960 8,980

GCTACAGCTACAGCTTCAATTATGCCACATGTTATGGAACAACCACTTTCAACATCAATTAATCCAGTTAATGATAGATTCAATGGAATTCCAAATCCAACACCATA
CGATGTCGATGTCGAAGTTAATACGGTGTACAATACCTTGTTGGTGAAAGTTGTAGTTAATTAGGTCAATTACTATCTAAGTTACCTTAAGGTTTAGGTTGTGGTAT

QF2w optimized

9,000 9,020 9,040 9,060 9,080

```
TAATTCAGATGCTGCTCTTGATGCTATTACACAAACAAATGATTATGGATCAGTTAATACACATGGAATTCTTTCAACATATCCACCACCAGCTACACATCTTAATG
ATTAAGTCTACGACGAGAACTACGATAATGTGTTTGTTTACTAATACCTAGTCAATTATGTGTACCTTAAGAAAGTTGTATAGGTGGTGGTCGATGTGTAGAATTAC
                                        QF2w optimized
   9,100          9,120          9,140          9,160          9,180          9,200

AAGCTTCAGTTGCTCTTGCTCCAGGAGGAGCTCCACCAAGACCACCACCACCATATGTTGATTCAACAACAAATCATCCACCATATCATTCAAATCTTGTTCCAATG
TTCGAAGTCAACGAGAACGAGGTCCTCCTCGAGGTGGTTCTGGTGGTGGTGGTATACAACTAAGTTGTTGTTTAGTAGGTGGTATAGTAAGTTTAGAACAAGGTTAC
                                        QF2w optimized
            9,220          9,240          9,260          9,280          9,300

GCTAATTTCGGATATTCAACAGTTGATTATGATGCTATGGTTGATGATCTTGCTTCAATTGAATATACAGATGCTGTTGATGTTGATCCACAATTCATGACAAATCT
CGATTAAAGCCTATAAGTTGTCAACTAATACTACGATACCAACTACTAGAACGAAGTTAACTTATATGTCTACGACAACTACAACTAGGTGTTAAGTACTGTTTAGA
                                        QF2w optimized
       9,320          9,340          9,360          9,380          9,400

                                                                   NotI
TGGATTCGTTCCAGGATGTAATTTCTCAGATATTAATACATATAAAAAAAAAAAATGAGCGGCCGCATTTTCAAATTTTAAATACTGAATATTTGTTTTTTTTCCTA
ACCTAAGCAAGGTCCTACATTAAAGAGTCTATAATTATGTATATTTTTTTTTTTTACTCGCCGGCGTAAAAGTTTAAAATTTATGACTTATAAACAAAAAAAAGGAT
                  QF2w optimized                                              let-858 3'UTR
  9,420          9,440          9,460          9,480          9,500          9,520

TTATTTATTTATTCTCTTTGTGTTTTTTTTCTTGCTTTCTAAAAAATTAATTCAATCCAAATCTAAacattttttttttctctttccgtctcccaattcgtattccgc
AATAAATAAATAAGAGAAACACAAAAAAAAGAACGAAAGATTTTTTAATTAAGTTAGGTTTAGATTtgtaaaaaaaaagagaaaggcagagggttaagcataaggcg
                                        let-858 3'UTR
           9,540          9,560          9,580          9,600          9,620

                    DraIII
tcctctcatctgaacacaatgtgcaagtttatttatcttctcgctttcatttcattaggacgtgggggaattggtggaagggggaaacacacaaaaggatgatgga
aggagagtagacttgtgttacacgttcaaataaatagaagagcgaaagtaaagtaatcctgcacccccttaaccaccttccccctttgtgtgttttcctactacct
                                        let-858 3'UTR
      9,640          9,660          9,680          9,700          9,720

                                                                                                       SpeI
aatgaaataaggacacacaatatgcaacaacattcaattcagaaatatggaggaaggtttaaaagaaaacataaaaatatatagaggaggaaggaaaactagtaaaa
ttactttattcctgtgtgttatacgttgttgtaagttaagtctttatacctccttccaaattttcttttgtatttttatatatctcctccttccttttgatcatttt
                                        let-858 3'UTR
  9,740          9,760          9,780          9,800          9,820          9,840

aataagcaaagaaattaggcgaacgatgAGAATTGTCCTCGCTTGGATAACTTCGTATAGCATACATTATACGAAGTTATACCGGAATTAATTCACAGCCACCGATC
ttattcgtttctttaatccgcttgctacTCTTAACAGGAGCGAACCTATTGAAGCATATCGTATGTAATATGCTTCAATATGGCCTTAATTAAGTGTCGGTGGCTAG
          let-858 3'UTR                      LoxP
             9,860          9,880          9,900          9,920          9,940
```

PaeR7I
XhoI
BssHII BsiWI TliI SbfI NcoI AgeI AvrII

TTCGAATGCATCGCGCGCACCGTACGTCTCGAGGAATTCCTGCAGGATATCTGGATCCACGAAGCTTCCCATGGTGACGTCACCGGTTCTAGATACCTAGGTGAGCT
AAGCTTACGTAGCGCGCGTGGCATGCAGAGCTCCTTAAGGACGTCCTATAGACCTAGGTGCTTCGAAGGGTACCACTGCAGTGGCCAAGATCTATGGATCCACTCGA

9,960 9,980 10,000 10,020 10,040

AflII AhdI

CTGGTACCCTCTAGTCAAGGCCTtaAGTGAGTCGTATTACGGactggccgtcgttttacacccgtgcagttgaaattgaaaatgagttaaagttggagtttttattg
GACCATGGGAGATCAGTTCCGGAatTCACTCAGCATAATGCCtgaccggcagcaaaatgtgggcacgtcaactttaactttttactcaatttcaacctcaaaaataac

M13-fwd CB unc-119 (+)

10,060 10,080 10,100 10,120 10,140 10,160

aaaacagatttccgtgtgattagtgtttttagcgagtgtgacaggacagcgaaaaaatatagaaacaagggggggaactgaaaagcttaggaatgcattgaacatgag
ttttgtctaaaggcacactaatcacaaaaatcgctcacactgtcctgtcgcttttttatatctttgttcccccccttgacttttcgaatccttacgtaacttgtactc

CB unc-119 (+)

10,180 10,200 10,220 10,240 10,260

aaggggaaggggaaggaacaaactagacaggaattattggaatttaatcacatttggagtttttttttctattcgacagaataattatccagaacatttttgtattaa
ttccccttccccttccttgtttgatctgtccttaataaccttaaattagtgtaaacctcaaaaaaaagataagctgtcttattaataggtcttgtaaaaacataatt

CB unc-119 (+)

10,280 10,300 10,320 10,340 10,360

atatttatgcatcatatgagtagtcggctttgttgtgcatgacgagtttgttatcgacgaaatagaagctgtcagaacgagtctcgtttggattgttgatcatgtcg
tataaatacgtagtatactcatcagccgaaacaacacgtactgctcaaacaatagctgctttatcttcgacagtcttgctcagagcaaacctaacaactagtacagc

CB unc-119 (+)

10,400 10,420 10,440 10,460 10,480

tccactgaaaaagagattagtcttttgaattgtacttttttagaataatgactcactgagttgttgagagagttgagggaactcatagatatgttcacagttgtttcg
aggtgactttttctctaatcagaaaacttaacatgaaaaatcttattactgagtgactcaacaactctctcaactcccttgagtatctatacaagtgtcaacaaagc

CB unc-119 (+)

10,500 10,520 10,540 10,560 10,580

tgaattcggaatacagaatccgaattcaaagtcaaaacacttcagaaggcgatctttgaagaagtgacgttcgatcattcggaaatgatggattgggatgtctccta
acttaagccttatgtcttaggcttaagtttcagttttgtgaagtcttccgctagaaacttcttcactgcaagctagtaagcctttactacctaaccctacagaggat

CB unc-119 (+)

10,600 10,620 10,640 10,660 10,680 10,700

ctttgaattccacagttgctccgaccgttttgagtttcaaaaagtttggagcaaatctataacgcacgtatcttgccgactcctgtggcgactcatcattctcctga
gaaacttaaggtgtcaacgaggctggcaaaactcaaagtttttcaaacctcgtttagatattgcgtgcatagaacggctgaggacaccgctgagtagtaagaggact

CB unc-119 (+)

10,720 10,740 10,760 10,780 10,800

tcgttctccggtttggcgatctcgaaaagcacttgctcagtgtccagatcacggatttggaacttggtgaactcgatgttatagatgttcgcagatggggagcataa
agcaagaggccaaaccgctagagcttttcgtgaacgagtcacaggtctagtgcctaaaccttgaaccacttgagctacaatatctacaagcgtctacccctcgtatt
CB unc-119 (+)
10,820 10,840 10,860 10,880 10,900

gaatcctaaatttatgttttaaactgaaatccaaagggagcaagataccttgagtgattcccggaagtgctaaaacgtcgttcggagtgatttgagctttcttcgca
cttaggatttaaatacaaaatttgactttaggtttccctcgttctatggaactcactaagggccttcacgattttgcagcaagcctcactaaactcgaaagaagcgt
CB unc-119 (+)
10,920 10,940 10,960 10,980 11,000 11,020

agctccgattccgttgtgattccttgttcggtgcttggtggtggccgtggcatctggaaatatggaaaagttcaacaaaaagaaaagagaaaagaatgaaatcggat
tcgaggctaaggcaacactaaggaacaagccacgaaccaccaccggcaccgtagacctttataccttttcaagttgtttttcttttctcttttcttactttagccta
CB unc-119 (+)
11,040 11,060 11,080 11,100 11,120

AarI
atcaagagttagttgagcggtttctctagttttctgagtctcacctgcgacgggaaggtcgccgagccgggtggaatcgatCgttgttgctcggctttcatatcggt
tagttctcaatcaactcgccaaagagatcaaaagactcagagtggacgctgcccttccagcggctcggcccaccttagctaGcaacaacgagccgaaagtatagcca
CB unc-119 (+)
11,140 11,160 11,180 11,200 11,220

ttggttggaagcggctgaaaacggaaagaagtggaagaaggaaaagagtgtggtgtgacaggaaaatggtaattagagggtgccaaataaccagctatattttgttt
aaccaaccttcgccgacttttgcctttcttcaccttcttccttttctcacaccacactgtccttttaccattaatctcccacggtttattggtcgatataaaacaaa
CB unc-119 (+)
11,240 11,260 11,280 11,300 11,320 11,340

BspEI
ttttttgaaaacatttttaaaaagaaaaatacgataatgatatcagatggatttccggaaaactggtatgaaaaatttcaacctttttgagtacatgtaatcaaaat
aaaaaactttttgtaaaaatttttctttttatgctattactatagtctacctaaaggccttttgaccatactttttaaagttggaaaaactcatgtacattagtttta
CB unc-119 (+)
11,360 11,380 11,400 11,420 11,440

SacII
acactttgtaaattatcattttttattgaaactccaccatttttctatttataacgctaataatttgaaaaagaaacctAttgcgaaccgcggggtgaatcccaaaaa
tgtgaaacatttaatagtaaaaataactttgaggtggtaaaaagataaatattgcgattattaaactttttctttggaTaacgcttggcgccccacttagggttttt
CB unc-119 (+)
11,460 11,480 11,500 11,520 11,540

cgaatgcgttttggtggagtgattgattcgaatcgaagaagaaaaagaagaagacgtggaatagagagctcactcttaaccgagcagcacacaccgacagaaaaaaa
gcttacgcaaaaccacctcactaactaagcttagcttcttctttttcttcttctgcaccttatctctcgagtgagaattggctcgtcgtgtgtggctgtcttttttt
CB unc-119 (+)
11,560 11,580 11,600 11,620 11,640 11,660

aatgaaatgaatgagggtcttcttcttcttcttcttcgaatgattgacagaaatgggaaaaagaggaagattgagaagggaaaaaggaaggagaaaagaagcagaag
ttactttacttactcccagaagaagaagaagaagaagcttactaactgtctttacccttttttctccttctaactcttcccttttttccttcctcttttcttcgtcttc
CB unc-119 (+)
11,680 11,700 11,720 11,740 11,760

aagacgtcagagaggagaggaacgagcggaaaagcagcgggcgcaagtcatagaagtagcagagctggggagaagaagacactatccaagaaaggaatgacgagaga
ttctgcagtctctcctctccttgctcgccttttcgtcgcccgcgttcagtatcttcatcgtctcgacccctcttcttctgtgataggttctttccttactgctctct
CB unc-119 (+)
11,780 11,800 11,820 11,840 11,860

gtatgcaaaggggtatagggtgcagacagaataggaacagaataacagatgatgagccaagaagagttgaaaagggcgatgaatttgtcatgtaacttaatttgggt
catacgtttccccatatcccacgtctgtcttatccttgtcttattgtctactactcggttcttctcaacttttcccgctacttaaacagtacattgaattaaaccca
CB unc-119 (+)
11,880 11,900 11,920 11,940 11,960 11,980

AgeI KasI NarI SfoI PluTI
caatttgagcatgatgaattgaaatcatcccttgttgggagttaataaccggtttgttatcagaaaccctgtaatagaagggcgccctaactttgagccaattcatc
gttaaactcgtactacttaactttagtagggaacaaccctcaattattggccaaacaatagtctttgggacattatcttcccgcgggattgaaactcggttaagtag
CB unc-119 (+)
12,000 12,020 12,040 12,060 12,080

HpaI
ccggtttctgtcaaatatatcaaaaagtggtcaactgacaaattgtttttgatattataataaacattttatccgttaacaattttcgaatactttttacaaggact
ggccaaagacagtttatatagtttttcaccagttgactgtttaacaaaaactataatattatttgtaaaataggcaattgttaaaagcttatgaaaaatgttcctga
CB unc-119 (+)
12,100 12,120 12,140 12,160 12,180

Tth111I PflFI
tggataaattggctcaAGCCTGCTTTTTTGTACaaacttgacgaagtgagtttgctaccatcatggaatcaggaggacatttcgacaatgtcatataatgtttgaaa
acctatttaaccgagtTCGGACGAAAAAACATGtttgaactgcttcactcaaacgatggtagtaccttagtcctcctgtaaagctgttacagtatattacaaacttt
attB1
Gateway recomb1 left recomb ttTi5605
12,200 12,220 12,240 12,260 12,280 12,300

aatgattttaacaagggtctcattcgagataagcaatgctctgagcaagtggctcgtcgtcttcaagaatctcttgttgatattcatttggatcctgttttgcacga
ttactaaaattgttcccagagtaagctctattcgttacgagactcgttcaccgagcagcagaagttcttagagaacaactataagtaaacctaggacaaaacgtgct
left recomb ttTi5605
12,320 12,340 12,360 12,380 12,400

NcoI

agcatgtgccatggatattcagagtttttgcaaaaaagttccgccaggtcaaacaatctttttaatttttatatgatacttcgggtgtatttttcttctttttcctg
tcgtacacggtacctataagtctcaaaaacgttttttcaaggcggtccagtttgttagaaaaattaaaaatatactatgaagcccacataaaaagaagaaaaaggac

left recomb ttTi5605

12,420 12,440 12,460 12,480 12,500

ttcattttcgctttttttctcactttttttgtaacaaaatctctcacttttctgtaagtcttaacaaaagttcaattggctcgatcaagtaaaatttcggccgtatgaa
aagtaaaagcgaaaaaagagtgaaaaaacattgttttagagagtgaaaagacattcagaattgttttcaagttaaccgagctagttcattttaaagccggcatactt

left recomb ttTi5605

12,540 12,560 12,580 12,600 12,620

cgttttcaaaggttttcatttttttttaagttcattcacaatatcatgaatctctcgtcctttggtttccggtaagtagcgaaataatataagcatacttataaatg
gcaaaagtttccaaaagtaaaaaaaaaattcaagtaagtgttatagtacttagagagcaggaaaccaaaggccattcatcgctttattatattcgtatgaatatttac

left recomb ttTi5605

12,640 12,660 12,680 12,700 12,720

aaggaattgaatatagaataaggaaagcataactaccaataacggaatatagaggtaatactgtaaatgtactgataaccaccattacagtattcattgaatatgcc
ttccttaacttatatcttattcctttcgtattgatggttattgccttatatctccattatgacatttacatgactattggtggtaatgtcataagtaacttatacgg

left recomb ttTi5605

12,740 12,760 12,780 12,800 12,820 12,840

BstXI

acagattgagcaatactacgatgtttttgaggtaccagttcagaggatataaaccacgaaattggaccaactccagttctgaaatttcattattcgattaaatcaaa
tgtctaactcgttatgatgctacaaaaactccatggtcaagtctcctatatttggtgctttaacctggttgaggtcaagactttaaagtaataagctaatttagttt

left recomb ttTi5605

12,860 12,880 12,900 12,920 12,940

SnaBI

atatatttgaaaaacctcacccataagtataaccgtagatgatcaatgcaactaggcatccgtatttcatcgggtcaatcagaatacgtatttttgcgaagaatacg
tatataaactttttggagtgggtattcatattggcatctactagttacgttgatccgtaggcataaagtagcccagttagtcttatgcataaaaacgcttcttatgc

left recomb ttTi5605

12,960 12,980 13,000 13,020 13,040

aatgcaattagagctaaattattgagaaatgtgaagagaaggagaagcttgcgtcgaccaactctgtaaacttattcatacaattttgtcttcaaattttgaacgtt
ttacgttaatctcgatttaataactctttacacttctcttcctcttcgaacgcagctggttgagacatttgaataagtatgttaaaacagaagtttaaaacttgcaa

left recomb ttTi5605

13,060 13,080 13,100 13,120 13,140 13,160

taaaactatcttattcaatgagttagcgggtttttatagtgagttagttcacgattatctaataaaactatgtccaaacatcctaactgcgaccgtattctcactaa
attttgatagaataagttactcaatcgcccaaaaatatcactcaatcaagtgctaatagattattttgatacaggtttgtaggattgacgctggcataagagtgatt

left recomb ttTi5605

13,180 13,200 13,220 13,240 13,260

ctcgtcttaaaaacgttgagattattttaaacccttcttcagtacaactacagtactgatgcacaaatacaataataataatacattcagacatactctgaacaatg
gagcagaatttttgcaactctaataaaatttgggaagaagtcatgttgatgtcatgactacgtgtttatgttattattattatgtaagtctgtatgagacttgttac
left recomb ttTi5605
13,280 13,300 13,320 13,340 13,360

taacttgtttttgttatactgtggtaacatttattgaaatgaaatataaccttacttttcaatcacaattccaccagtaattgttccagaaacataagctaaaccca
attgaacaaaaacaatatgacaccattgtaaataactttactttatattggaatgaaaagttagtgttaaggtggtcattaacaaggtctttgtattcgatttgggt
left recomb ttTi5605
13,380 13,400 13,420 13,440 13,460 13,480

ttgcagttgtactccattcagcaatttctgactcaacatttgcattttctagaaagaaagtagaggataaaagaagagcccataatccgacaagcaacttttctata
aacgtcaacatgaggtaagtcgttaaagactgagttgtaaacgtaaaagatctttctttcatctcctattttcttctcgggtattaggctgttcgttgaaaagatat
left recomb ttTi5605
attB4
13,500 13,520 13,540 13,560 13,580

caaagttgatagcttggcgGATccctggcgttacccaacttaatcgccttgcagcacatccccctttcgccagctggcgtaatagcgaagaggcccgcaccgatcgc
gtttcaactatcgaaccgcCTAgggaccgcaatgggttgaattagcggaacgtcgtgtagggggaaagcggtcgaccgcattatcgcttctccgggcgtggctagcg
LacZ alpha
13,600 13,620 13,640 13,660 13,680

PluTI
SfoI
NarI
KasI
ccttcccaacagttgcgcagcctgaatggcgaatggcgcctgatgcggtattttctccttacgcatctgtgcggtatttcacaccgcatatggtgcactctcagtac
ggaagggttgtcaacgcgtcggacttaccgcttaccgcggactacgccataaaagaggaatgcgtagacacgccataaagtgtggcgtataccacgtgagagtcatg
LacZ alpha
13,700 13,720 13,740 13,760 13,780 13,800

aatctgctctgatgccgcatagttaagccagccccgacacccgccaacacccgctgacgcgccctgacgggcttgtctgctcccggcatccgcttacagacaagctg
ttagacgagactacggcgtatcaattcggtcggggctgtgggcggttgtgggcgactgcgcgggactgcccgaacagacgagggccgtaggcgaatgtctgttcgac
13,820 13,840 13,860 13,880 13,900

tgaccgtctccgggagctgcatgtgtcagaggttttcaccgtcatcaccgaaacgcgcgagacgaaagggcctcgtgatacgcctatttttataggttaatgtcatg
actggcagaggccctcgacgtacacagtctccaaaagtggcagtagtggctttgcgcgctctgctttcccggagcactatgcggataaaaatatccaattacagtac
13,920 13,940 13,960 13,980 14,000

ataataatggtttcttagacgtcaggtggcacttttcggggaaatgtgcgcggaacccctatttgtttatttttctaaatacattcaaatatgtatccgctcatgag
tattattaccaaagaatctgcagtccaccgtgaaaagcccctttacacgcgccttggggataaacaaataaaaagatttatgtaagtttatacataggcgagtactc
AmpR promoter
14,020 14,040 14,060 14,080 14,100 14,120

acaataaccctgataaatgcttcaataatattgaaaaaggaagagtatgagtattcaacatttccgtgtcgcccttattcccttttttgcggcattttgccttcctg
tgttattgggactatttacgaagttattataactttttccttctcatactcataagttgtaaaggcacagcgggaataagggaaaaaacgccgtaaaacggaaggac

AmpR promoter

AmpR

14,140 14,160 14,180 14,200 14,220

tttttgctcacccagaaacgctggtgaaagtaaaagatgctgaagatcagttgggtgcacgagtgggttacatcgaactggatctcaacagcggtaagatccttgag
aaaaacgagtgggtctttgcgaccactttcattttctacgacttctagtcaacccacgtgctcacccaatgtagcttgacctagagttgtcgccattctaggaactc

AmpR

14,240 14,260 14,280 14,300 14,320

agttttcgccccgaagaacgttttccaatg
tcaaaagcggggcttcttgcaaaaggttac

AmpR

14,340 14,350 14,360
